# Lactate-mediated NK cell dysfunction as a prognostic marker and therapeutic target in breast cancer

**DOI:** 10.1038/s41420-026-03063-5

**Published:** 2026-03-27

**Authors:** Simone Ielpo, Francesca Barberini, Alice Gaiba, Camilla Baronti, Marco Greppi, Valentina Obino, Silvia Ravera, Nicole Bussola, Adele De Ninno, Luca Businaro, Valentina Mussi, Silvia Pomella, Emanuele Agolini, Monica Benvenuto, Chiara Focaccetti, Emanuela Marcenaro, Roberto Bei, Giovanni Barillari, Silvia Pesce, Loredana Cifaldi, Ombretta Melaiu

**Affiliations:** 1 Department of Clinical Sciences and Translational Medicine, University of Rome Tor Vergata, Rome, Italy; 2 Department of Experimental Medicine (DIMES), University of Genoa, Genoa, Italy; 3 IRCCS AOM (Azienda Ospedaliera Metropolitana), Genoa, Italy; 4 Icahn School of Medicine at Mount Sinai, New York, NY USA; 5 Institute for Photonics and Nanotechnology, Italian National Research Council, Rome, Italy; 6 Institute for Microelectronics and Microsystems, Italian National Research Council, Rome, Italy; 7 Department of Onco-Haematology and Cell and Gene Therapy, Bambino Gesù Children’s Hospital, IRCCS, Rome, Italy; 8 Laboratory of Medical Genetics, Translational Cytogenomics Research Unit, Bambino Gesù Children Hospital, IRCCS, Rome, Italy

**Keywords:** Immunosurveillance, Translational research, Breast cancer, Innate lymphoid cells

## Abstract

Lactate is recognized as a crucial signalling molecule within the tumor microenvironment, where it shapes immune responses by modulating various cell populations, including T cells and macrophages. However, its effect on natural killer (NK) cells, key effectors of early antitumor immunity, remains poorly understood. This study investigates how intratumoral lactate accumulation affects NK cell function in breast cancer, a neoplasm characterized by elevated glycolytic flux. An in-silico analysis of 882 breast cancer patients revealed that high lactate metabolism is inversely correlated with NK cell activation genes and is associated with poor prognosis. To corroborate these findings, NK cells from healthy donors were cultured under lactate-rich or control conditions. Lactate exposure impaired NK cell proliferation, downregulated activation markers and cytotoxic molecules, disrupted mitochondrial bioenergetics, and induced lipid accumulation, as demonstrated by flow cytometry, metabolic profiling, and Raman spectroscopy. Functional assays using microfluidic devices and degranulation tests revealed that lactate-exposed NK cells exhibited reduced chemotaxis and diminished cytotoxicity against MCF-7 and MDA-MB-231 breast cancer spheroids, accompanied by decreased CXCL9 and CXCL10 production. Pharmacologic inhibition of lactate transport, via Syrosingopine or MSC-4381 and AZD3965 combination, restored NK cell cytotoxicity in tumor co-cultures, as shown by increased NK cell degranulation, caspase-3/7–mediated tumor apoptosis, and spheroid shrinkage. Finally, GPR81 deletion mirrored these effects, enhancing NK cell activity. These findings identify lactate as a driver of NK cell suppression and highlight lactate transport and receptor targeting as a strategy to enhance NK cell–based immunotherapies in breast cancer and other lactate-rich tumors.

## Introduction

Metabolic reprogramming is a hallmark of cancer, enabling malignant cells to sustain rapid proliferation, adapt to stress, and evade immune surveillance [1]. A central feature of this reprogramming is the Warburg phenotype, characterized by elevated glucose uptake and preferential conversion of pyruvate to lactate even under normoxic conditions [2]. First described by Otto Warburg in the 1920s, this form of aerobic glycolysis is observed across diverse tumor types and is associated with poor prognosis and limited therapeutic response [3]. Despite its lower energetic yield compared to oxidative phosphorylation (OxPhos), the Warburg phenotype confers several advantages to cancer cells, including continuous glycolytic flux, provision of biosynthetic precursors, and modulation of the tumor microenvironment (TME) [4].

Efficient lactate export is essential to sustain glycolysis and prevent intracellular acidification. This process is mediated primarily by the proton-coupled monocarboxylate transporters (MCT) 1 and 4, which co-transport lactate and protons into the extracellular space [5]. The consequent lactate accumulation and acidification of the TME are not passive waste-disposal processes, but active drivers of tumor progression [6]. Lactate also acts as an immunomodulatory metabolite, shaping the immune landscape to favor tumor persistence [7].

The emerging field of immunometabolism highlights the tight link between immune cell function and cellular metabolic pathways [8]. Activation of immune effector cells typically involves a shift from OxPhos to glycolysis to support the increased demand for biosynthetic substrates [9]. This metabolic flexibility is influenced by nutrient availability, signalling pathways, oxygen tension, and extracellular pH. Within the TME, however, hypoxia, nutrient deprivation, and the accumulation of metabolites such as lactate impair immune cell metabolism and drive their differentiation toward immunosuppressive phenotypes, ultimately fostering immune tolerance and promoting cancer progression [10].

Natural killer (NK) cells are highly sensitive to the metabolic composition of the TME [11]. These innate lymphoid cells play a critical role in tumor surveillance by detecting and eliminating transformed or stressed cells without prior antigen sensitization [12]. Their antitumor function relies on direct cytotoxicity, primarily via perforin/granzyme release, and through production of cytokines such as interferon-γ (IFN-γ), which modulate adaptive immune responses [13]. Tumor-derived metabolites, including lactate, have been shown to directly impair NK cell function, contributing to disease progression in melanoma, pancreatic cancer, and colorectal liver metastases [14–16]. Mechanistic studies indicated that a hypoxic TME can alter NK cell metabolism by inducing mitochondrial fragmentation [17], while lactate can promote protein lactylation, suppressing cytotoxic activity against leukemia cells [18]. However, how lactate alters NK cell function in breast cancer, and the resulting functional consequences, remain unclear. This gap prompted us to explore the specific role of this metabolic–immunologic axis in this disease context. Breast cancer provides an especially relevant model, as higher tumor grade correlates with elevated intratumoral lactate levels, and increased lactate dehydrogenase (LDH) expression is linked to poor clinical outcomes [19–21].

In this study, we examined the clinical impact of tumor-derived lactate on NK cell function against breast cancer. We demonstrated that high lactate metabolism inversely correlated with NK cell activation gene signature and predicted unfavorable patient outcomes. Functional studies and co-culture experiments revealed that lactate exposure impairs NK cell activation, cytotoxicity, metabolic fitness, and chemotaxis. Importantly, pharmacological inhibition of lactate transport using either the dual MCT inhibitor Syrosingopine or combined MSC-4381 and AZD3965 treatment restored NK cell cytotoxicity, reduced tumor-derived lactate secretion, and alleviated TME acidification. Notably, deletion of the GPR81 lactate receptor promotes a TME that enhances NK cell activity.

Collectively, our findings identify actionable metabolic target to overcome lactate-mediated NK cell suppression, offering new avenues for therapeutic intervention in breast cancer and other lactate-rich malignancies.

## Results

### Inverse association between genes involved in lactate metabolism and NK cell activation predicts unfavorable prognosis of breast cancer patients

To explore the potential relationship between the lactate metabolic pathway and NK cell function, we analyzed transcriptomic data from 882 breast cancer patients (GSE115577). We focused on the expression of genes specifically expressed by NK cells, including *NCR1*, *NCR2*, and *NCR3*, which encode the activating receptors NKp46, NKp30, and NKp44, as well as genes involved in lactate metabolism, such as *LDHA* which encode lactate dehydrogenase A, an enzyme that catalyzes the reversible conversion of pyruvate to lactate, and *HCAR1* encoding hydroxycarboxylic acid receptor 1, a G-protein-coupled receptor activated by L-lactate.

**Fig. 1:**
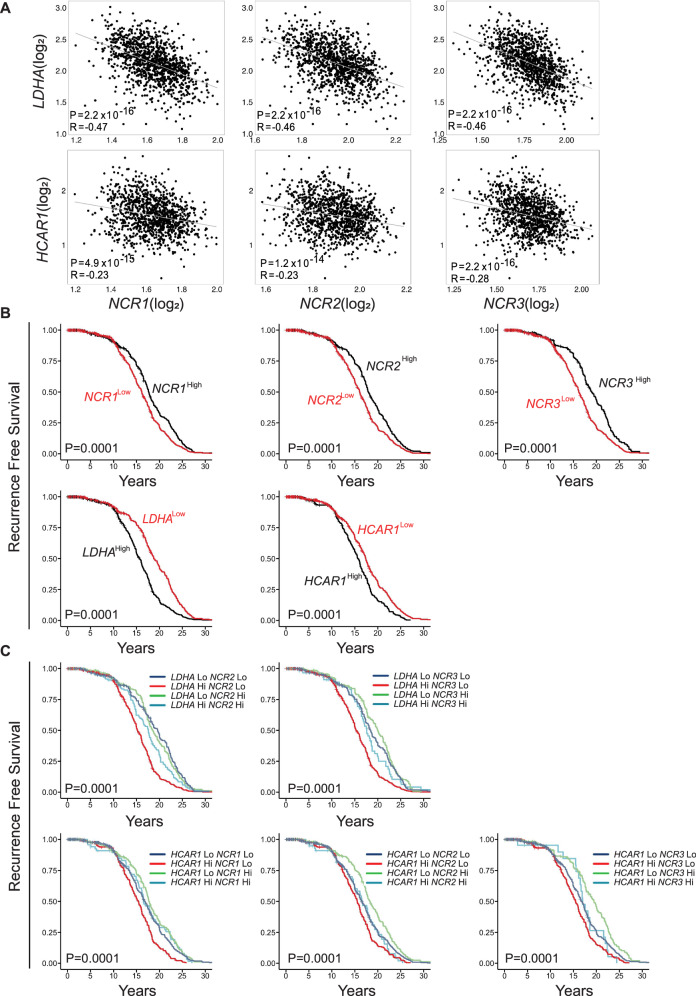
Inverse relationship between lactate metabolism–related genes and NK cell activity and their impact on clinical outcomes in breast cancer patients. **A** Scatter plots showing significant negative correlations between *LDHA* (top) or *HCAR1* (bottom) and NK cell receptor genes (*NCR1*, *NCR2*, *NCR3*) in the *GSE115577* breast cancer dataset (*n* = 882). Pearson correlation coefficients (*R*) and *p*-values are indicated. **B** Kaplan–Meier curves depicting recurrence-free survival of breast cancer patients stratified by high and low expression of *NCR1*, *NCR2*, and *NCR3* alone (top) or *LDHA* and *HCAR1* alone (bottom). **C** Kaplan–Meier curves showing combined survival analysis stratified by *LDHA* or *HCAR1* expression and NK cell receptor expression (*NCR1*, *NCR2*, *NCR3*). In (**B**, **C**), log-rank tests with Miller and Siegmund *p*-value correction were applied to the *GSE115577* breast cancer dataset (*n* = 882). Exact *p*-values are reported in the corresponding plot.

The expression of both *LDHA* and *HCAR1* showed strong negative correlations with *NCR1*, *NCR2*, and *NCR3*, thus suggesting that the elevated expression of lactate-related genes is associated with suppressed NK cell activation signatures in breast cancer patients (Fig. 1A). Given this strong inverse association, we next evaluated the prognostic significance of NK cell activity in a lactate-rich TME.

Kaplan–Meier survival analyses revealed that high expression of NK cell-activating receptors (*NCR1*, *NCR2*, *NCR3*) was significantly associated with improved recurrence-free survival. In contrast, high expression of *LDHA* or *HCAR1* was correlated with poorer prognosis (Fig. 1B). Notably, when combining expression profiles, patients with high *LDHA* and low *NCR2/3* expression exhibited the worst survival outcomes, whereas those with low *LDHA* and high NK cell receptor expression had the most favorable prognosis. A similar pattern was observed for *HCAR1*, where high *HCAR1* expression combined with low expression of the NK cell receptor was associated with reduced recurrence-free survival (Fig. 1C).

To refine these observations, multivariable survival analyses were performed using Cox proportional hazards models when the proportional hazards assumption was met and restricted mean survival time (RMST) analyses otherwise, adjusting for age at diagnosis, tumor grade, HER2 status, ER status, and tumor size. In these models, high expression of *NCR1*, *NCR2*, and *NCR3* was associated with significantly longer risk of recurrence, with ΔRMST estimates ranging from 1.61 to 2.41 years relative to low expression (*p*-value < 0.001 for all comparisons). Conversely, low *HCAR1* expression was associated with a significantly reduced risk of recurrence compared with high expression (HR = 0.68, 95% CI [0.59, 0.79], *p*-value < 0.001) (Supplementary Table 1). In Cox proportional hazards models for *HCAR1*–*NCR1* and *HCAR1*–*NCR2*, tumors with high *HCAR1* expression combined with low NCR expression exhibited markedly increased recurrence risk compared with the reference group (HR [1.81, 1.88], *p*-value < 0.001). Intermediate joint expression states showed attenuated effects. For the *HCAR1*–*NCR3* combination, the proportional hazards assumption was violated, and RMST analysis demonstrated a substantial increase in recurrence-free survival with low *HCAR1* and high *NCR3* expression (ΔRMST = 3.19 years, 95% CI [2.22, 4.16], *p*-value = 1.15 × 10^−10^) (Supplementary Table 1).

Analogous analyses using *LDHA* yielded highly consistent results. In single-gene models, high *LDHA* expression was associated with significantly shorter recurrence free survival (RFS, ΔRMST = −3 years, *p*-value < 0.001). Joint *LDHA*–NCR models revealed a robust pattern across all NCRs: tumors with low *LDHA* expression combined with high NCR expression conferred the most favorable outcomes (ΔRMST range 2.19–3.6 years) (Supplementary Table 1).

Collectively, these findings suggest that the upregulation of the lactate metabolic axis (*LDHA*/*HCAR1*) is associated with the downregulation of NK cell activating receptors and correlates with poorer clinical outcomes in breast cancer patients. These findings underscore a potential immunosuppressive role of the lactate pathway, which may specifically impair NK cell-mediated antitumor surveillance.

### Lactate exposure inhibits NK cell proliferation, activation, and cytotoxicity in breast cancer

Next, we assessed the effect of lactate on the phenotypic and functional behaviour of NK cells in the context of breast cancer. As model system, we selected two breast cancer cell lines: MCF-7, representing a less aggressive luminal-A subtype, and MDA-MB-231, derived from a patient with triple-negative breast cancer, one of the most aggressive forms of the disease. Both models accurately recapitulate the in vivo lactate landscape in breast cancer, which rises with tumor aggressiveness, as reflected by increasing plasma lactate levels across breast cancer patients with different histotypes (Supplementary Fig. S1A).

Western blot and immunocytochemistry analyses revealed that both cell lines express the lactate transporters MCT1 and MCT4 (Supplementary Fig. S1B, C) as well as the lactate receptor GPR81 (encoded by the *HCAR1* gene) (Supplementary Fig. S1D). Consistent with the Warburg phenotype, significant lactate secretion was detected in the culture supernatant of both cell lines (Supplementary Fig. S1E).

To determine whether NK cells possess similar metabolic machinery, the expression of MCT1, MCT4, and GPR81 was profiled across primary human NK cells from different donors. Western blot and immunocytochemistry analyses revealed that NK cells express both lactate transporters, MCT1 and MCT4, as well as the lactate receptor GPR81. Although inter-donor variability was observed, all samples exhibited significant levels of these key lactate regulators (Supplementary Fig. S1F–H).

**Fig. 2:**
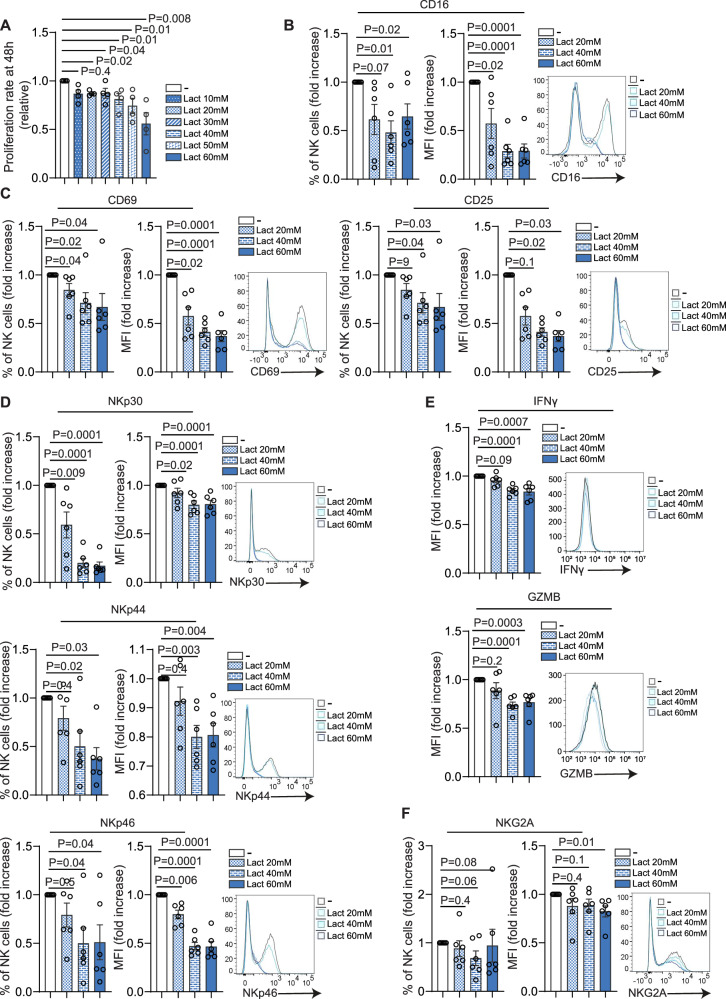
Effects of increasing lactate concentrations on NK cell viability, phenotype, and function. **A** NK cell proliferation rate after 48 h incubation with the indicated concentrations of sodium L-lactate (Lact) or without lactate (-). Data represent mean ± SEM from independent biological replicates performed with NK cells derived from four independent donors. Each dot represents one donor (*n* = 4). Statistical significance was determined using one-way ANOVA followed by Dunn’s multiple-comparison test. Exact *p*-values are reported in the plot. Expression of **B** Fc receptor CD16 (*n* = 6), **C** activation markers CD69 (*n* = 6) and CD25 (*n* = 6), **D** natural cytotoxicity receptors NKp30 (*n* = 6), NKp44 (*n* = 6), and NKp46 (*n* = 6), **E** cytotoxic molecules IFN-γ (*n* = 6) and granzyme B (GZMB, *n* = 6), and **F** inhibitory receptor NKG2A (*n* = 6) on NK cells cultured with the indicated concentrations of lactate (Lact) or without lactate (−). Data are shown as both percentage of positive cells (left for **B**, **C**, **D**, **F**) and MFI fold change (right) relative to untreated controls. Representative flow-cytometry histograms depicting MFI shifts for each marker are also shown. Data represent mean ± SEM from independent biological replicates using NK cells from six healthy donors (*n* = 6); each dot represents one donor. Statistical significance was assessed using one-way ANOVA followed by Dunn’s multiple-comparison test; exact *p*-values are reported in the corresponding plots.

Recent studies have highlighted lactate as a major metabolic component of the TME, particularly within hypoxic regions of solid tumors where oxygen availability is limited [22]. Building on this evidence, we investigated the direct impact of a lactate-rich TME by exposing primary NK cells in vitro to increasing concentrations of sodium L-lactate (hereafter referred to as lactate) and assessing their functional properties. First, a dose-response proliferation analysis across a wide range of lactate concentrations (10–60 mM) was performed to define the threshold at which lactate begins to impair NK cell function. At 24 h, NK cell proliferation was largely preserved at all tested concentrations (Supplementary Fig. S2A). In contrast, by 48 h lactate exposure induced a clear dose-dependent reduction in NK cell proliferation, with relatively modest effects at lower concentrations and progressively stronger inhibition at higher lactate levels (Fig. 2A). Specifically, proliferation was significantly reduced by approximately 13% at 10–30 mM, ~20% at 40 mM, and further decreased by ~26% and ~44% at 50 and 60 mM lactate, respectively.

Next, selected lactate concentrations (20, 40, and 60 mM) were used to examine their effects on NK cell phenotypic profile. Exposure to 40 and 60 mM lactate induced highly comparable phenotypic and functional alterations across multiple NK cell markers, indicating that near-maximal effects are already achieved at 40 mM. In contrast, 20 mM lactate exerted a more modest and variable impact, with partial or non-significant changes depending on the parameter analyzed, thus supporting a dose-dependent response with a clear threshold between 20 and 40 mM.

Specifically, both the frequency and mean fluorescence intensity (MFI) of CD16⁺ NK cells were progressively reduced following exposure to 20–60 mM lactate (Fig. 2B). Similarly, the frequency and MFI of the early activation markers CD69 and CD25 were significantly diminished, with more pronounced effects at 40 and 60 mM, indicating an overall reduction in NK cell activation status (Fig. 2C). In line with transcriptomic findings (Fig. 1), lactate exposure also significantly reduced the frequency and MFI of NKp30⁺, NKp44⁺, and NKp46⁺ NK cells (Fig. 2D). These effects were particularly evident at 40 and 60 mM, although a milder but significant reduction was already detectable at 20 mM. Furthermore, intracellular staining revealed that lactate impaired the production of key cytotoxic mediators, including IFN-γ and granzyme B (GZMB), as demonstrated by a significant reduction in their MFI at 40 and 60 mM but not at 20 mM (Fig. 2E). These findings confirm lactate-induced suppression of NK cell cytotoxic potential, already detectable at low concentrations and enhanced at higher levels. No significant changes were observed in the expression of CD57, a marker of mature and terminally differentiated NK cells, across all lactate concentrations tested (Supplementary Fig. S2B). Among inhibitory receptors, lactate exposure selectively reduced NKG2A MFI at 60 mM without significantly affecting the frequency of NKG2A⁺ NK cells at any dose (Fig. 2F), whereas the expression of the inhibitory receptors KIR3DL1 and KIR3DL2 does not undergo significant modulation at any lactate concentration tested (Supplementary Fig. S2C).

In breast cancer, intratumoral lactate concentrations can reach or exceed 50 mM, particularly in poorly perfused and necrotic tumor niches [3, 23]. Notably, despite the pronounced anti-proliferative effects observed at high lactate concentrations (Fig. 2A), lactate exposure did not induce overt cytotoxicity. Annexin V/7-AAD staining revealed no significant increase in early or late apoptotic NK cell populations at either 24 or 48 h following exposure to 60 mM lactate (Supplementary Fig. S2D, E). Overall cell viability remained comparable to untreated controls (Supplementary Fig. S2D, E), indicating that high lactate imposes a sublethal metabolic stress rather than inducing cell death. Based on these observations, we selected 60 mM lactate for subsequent experiments to mimic extreme yet biologically relevant tumor microenvironmental conditions, while acknowledging that lower lactate concentrations are sufficient to significantly impair NK cell functionality within the TME.

### Lactate affects NK cell function independently of extracellular and intracellular acidification

To determine whether the effects of lactate on NK cells were attributable to acidification or to lactate itself, we employed pH-matched acidic control conditions and performed direct, quantitative measurements of both extracellular pH over time and intracellular pH after 48 h of treatment (Supplementary Fig. S3). The lactate-containing medium maintained a stable, neutral extracellular pH comparable to that of the control medium (mean pH: 7.674; maximum: 7.714; minimum: 7.612 vs mean pH: 7.638; maximum: 7.670; minimum: 7.587 respectively), consistent with the use of sodium L-lactate and the buffering capacity of RPMI medium under controlled CO₂ conditions, and was distinct from the acidic condition (mean pH: 6.432; maximum: 6.445; minimum: 6.419) (Supplementary Fig. S3A, B). Intracellular pH measurements mirrored these findings. Both fluorescence microscopy and flow cytometry analyses revealed comparable intracellular pH levels in NK cells cultured in lactate-containing and control media, whereas exposure to acidic medium resulted in a marked increase in pHrodo fluorescence, indicative of significant intracellular acidification (Supplementary Fig. S3C, D).

In line with previous observations (Fig. 2A), NK cell proliferation at 48 h was significantly reduced by 60 mM lactate without affecting cell viability, supporting a cytostatic rather than cytotoxic effect (Supplementary Fig. S3E, F). In contrast, acidic pH induced a more severe suppression of proliferation accompanied by overt signs of cellular distress, consistent with non-specific, acid-mediated toxicity (Supplementary Fig. S3E, F).

Together, these findings demonstrate that lactate-induced NK cell dysfunction occurs independently of extracellular and intracellular pH alterations.

### Lactate alters the metabolic fingerprint of NK cells and impairs their mitochondrial bioenergetics

**Fig. 3:**
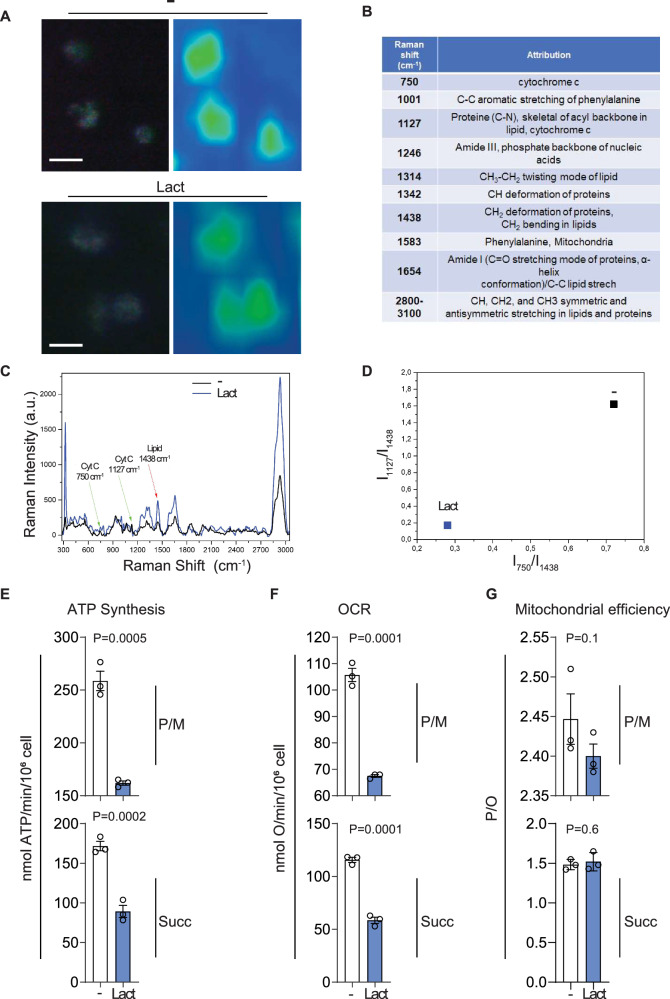
Effects of lactate on NK cell mitochondrial function and bioenergetics in breast cancer. **A** Representative chemical Raman images of NK cells cultured with (Lact) or without (−) lactate. **B** Assignment of the most relevant Raman contributions that differs in NK cells when cultured with or without lactate. **C** Raman spectra highlighting modifications induced in NK cells after culture in the presence of Lactate. **D** Ratios I₇₅₀/I₁₄₃₈ and I₁₁₂₇/I₁₄₃₈, reflecting Raman intensities of reduced cytochrome c (750, 1127 cm⁻¹) relative to lipids (1438 cm⁻¹), as an index of respiratory activity versus lipid metabolism. **E** ATP synthesis through FoF1-ATP synthase. **F** Oxygen consumption rate (OCR). **G** P/O value, an OxPhos efficiency marker. In **E**–**G**, the analyses were conducted on NK cells in the presence or absence of lactate, using pyruvate plus malate (P/M) or succinate (Succ) to induce the OxPhos pathways led by Complex I or Complex II, respectively. In **E**–**G**, data represent mean ± SEM from independent biological replicates performed with NK cells derived from three different healthy donors (*n* = 3). Each dot represents one donor. Statistical significance was determined using a two-tailed Student’s *t*-test. Exact *p*-values are reported in the corresponding plots.

To determine whether lactate alters the metabolic fingerprint of NK cells, we employed Raman spectroscopy, a label-free and non-destructive valuable tool capable of revealing metabolic changes in live cells [24]. Representative Raman images of NK cells cultured in the absence or presence of lactate, obtained using 532 nm excitation, showed no significant differences in cell size or morphology (Fig. 3A). In contrast, the corresponding Raman spectra, providing detailed structural insights into subcellular components, revealed distinct differences in the biochemical composition of NK cells exposed to lactate compared to controls (Fig. 3B,C). Specifically, spectral changes were observed in peaks associated with proteins, lipids, and cytochromes. The peak at 1001 cm^−1^ attributed to phenylalanine ring breathing [25], while the two other prominent peaks at 750 and 1127 cm^−1^ correspond to reduced cytochromes. Bands at 1342 cm^−1^ and 1438 cm^−1^ are attributed to C–H deformations in proteins and lipids. The band at 1654 cm⁻¹ represents an overlap of signals from protein amide I modes and C=C stretching in lipids [25]. Peaks at 2800–3100 cm^−1^ are associated with CH, CH₂, and CH₃ stretching modes in both lipids and proteins [25]. The spectral peak assignments are summarized in Fig. 3B. To quantify the balance between respiratory activity and lipid metabolism, the ratios I₇₅₀/I₁₄₃₈ and I₁₁₂₇/I₁₄₃₈ (cytochrome c to lipid signal) were calculated. These ratios were reduced in lactate-exposed NK cells, indicating lower levels of reduced cytochrome c and increased lipid accumulation compared to controls (Fig. 3C, D).

Given these profound changes in the biochemical fingerprint of lactate exposed NK cells, we next evaluated mitochondrial function through direct metabolic assays. Specifically, ATP production via FoF₁-ATP synthase, OCR, and OxPhos efficiency were assessed in NK cells cultured with or without lactate for 48 h. Data revealed that NK cells exposed to lactate exhibited impaired ATP production (Fig. 3E) and reduced OCR (Fig. 3F) following stimulation with both pyruvate/malate (P/M) and succinate, suggesting broad mitochondrial dysfunction [26].

Evaluation of mitochondrial coupling efficiency, as measured by the P/O ratio (ATP produced per oxygen atom consumed), revealed a decrease following P/M stimulation, but not with succinate, in lactate-treated NK cells (Fig. 3G). This indicates a specific impairment of OxPhos efficiency via complexes I–III–IV, suggesting not only reduced mitochondrial output but also compromised energy conversion efficiency [27].

Collectively, these findings demonstrate that lactate disrupts mitochondrial metabolism in NK cells, resulting in reduced OxPhos, diminished ATP production, and impaired mitochondrial efficiency. This metabolic suppression likely contributes to the observed functional impairment of NK cells, as insufficient energy availability may hinder their cytotoxic potential in the TME [28].

### Lactate impairs NK cell recruitment and function by suppressing chemotactic signalling and cytotoxic activation against breast cancer spheroids

**Fig. 4:**
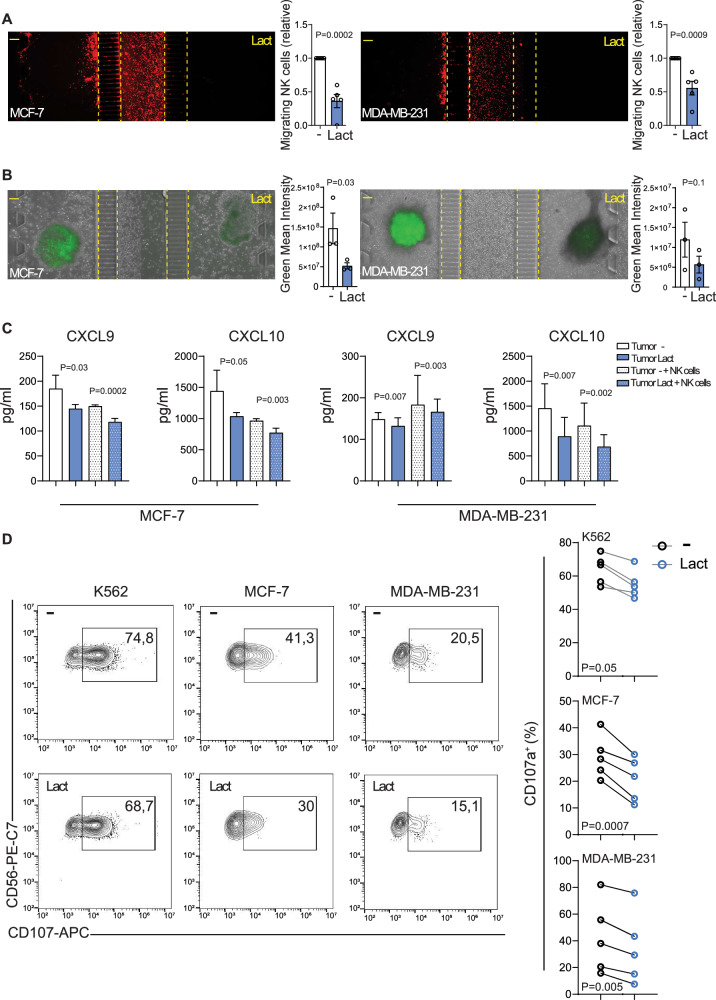
Lactate-driven suppression of NK cell antitumor functions. **A** Representative images of red-labeled NK cell migration in microfluidic devices toward MCF-7 (left) or MDA-MB-231 (right) tumor spheroids cultured in lactate-poor (left chamber) or lactate-rich (right chamber) environments for 48 h. The number of NK cells migrating toward each side of the device was quantified using ImageJ software. Data are shown as fold change ± SEM from independent biological replicates performed with NK cells derived from five different healthy donors (*n* = 5). Each dot represents one donor. Statistical significance was assessed using a two-tailed Student’s *t*-test, and *p*-values are reported in the individual plots. **B** Representative images of caspase-3/7–positive MCF-7 (left) or MDA-MB-231 (right) tumor spheroids after 48 h of co-culture with NK cells within microfluidic devices in lactate-poor (device left chamber) or lactate-rich (device right chamber) environments. Quantification of green fluorescence intensity, depicting the level of tumor apoptosis induced by NK cells under each experimental condition, was performed using ImageJ software. Data are expressed as mean ± SEM from three technical replicates and are representative of one of three independent biological experiments performed with NK cells derived from three different healthy donors (*n* = 3). Statistical significance was assessed using a two-tailed Student’s *t*-test, and exact *p*-values are reported in the individual plots. **C** Levels of the indicated chemokines measured in MCF-7 (left) and MDA-MB-231 (right) tumor spheroids cultured alone or co-cultured with NK cells in lactate-poor or lactate-rich environments. Bars represent mean ± SEM from one experiment representative of three independent biological experiments (*n* = 3). Statistical significance was assessed using one-way ANOVA followed by Dunn’s multiple-comparison test; exact *p*-values are reported in the corresponding plots. **D** Left, representative example of degranulation by human CD45^+^CD56^+^CD16^+^CD3^−^ NK cells from a healthy donor, either exposed or not exposed to lactate, measured as CD107a cell-surface expression following stimulation with K562, MCF-7, or MDA-MB-231 target cells. The percentage of CD107a⁺ NK cells is indicated. A summary of NK cell degranulation after stimulation with the indicated target cells from five healthy donors is shown on the right. Each dot represents NK cells from one donor (*n* = 5). Statistical significance was assessed using a two-tailed Student’s *t*-test. Exact *p*-values are reported in the corresponding plots.

To assess the impact of lactate on NK cell recruitment toward tumor spheroids, we employed microfluidic co-culture devices [29, 30]. Red-labelled NK cells derived from healthy donors were introduced into the central chamber of the devices, while spheroids derived from MCF-7 to MDA-MB-231 cell lines were placed in the side chambers, either with (right) or without (left) the addition of lactate. Over 48 h, NK cells exhibited significantly greater migration toward spheroids without lactate supplementation, with markedly reduced recruitment toward those exposed to lactate. This inhibitory effect was observed in co-cultures with both MCF-7 and MDA-MB-231 spheroids (Fig. 4A).

Consistent with reduced NK cell recruitment, apoptotic activity within tumor spheroids, measured using a green luminescent caspase 3/7 detection reagent, was significantly higher under lactate-free conditions (left channel). In contrast, spheroids exposed to lactate (right channel) exhibited minimal apoptosis, suggesting that the few NK cells able to infiltrate under these conditions were also functionally impaired. This effect was more pronounced in MCF-7 co-cultures but was also evident in those with MDA-MB-231 (Fig. 4B), aligning with previously observed reductions in cytotoxic mediators such as IFN-γ and GZMB (Fig. 2F).

To gain mechanistic insight, we measured the levels of two key chemokines involved in immune cell recruitment and activation, CXCL9 and CXCL10, both of which play pivotal roles in NK cell trafficking [31]. Lactate suppressed the secretion of both CXCL9 and CXCL10 in MCF-7 and MDA-MB-231 cancer spheroids, both in monoculture and more markedly during co-culture with NK cells (Fig. 4C), highlighting its role in impairing NK cell recruitment by downregulating essential chemotactic signals.

To further investigate the interaction between MCF-7 and MDA-MB-231 tumor cells and NK cells under lactate-rich conditions, we first examined the expression profiles of surface ligands involved in NK cell activation and inhibition (Supplementary Fig. S4). Apart from the epithelial cell adhesion molecule (EpCAM), which is abundantly expressed in MCF-7 cells but only weakly in MDA-MB-231 cells, both cell lines showed low expression of CD90 and CD133. In contrast, both MCF-7 and MDA-MB-231 cells expressed high levels of multiple ligands known to interact with NK cell receptors, including HLA class I molecules (HLA-I), Poliovirus receptor (PVR/CD155), Nectin-2 (CD112), and the stress-induced ligands ULBP and MICA/B. MCF-7 cells displayed low expression of the immune checkpoint ligands PD-L1 and PD-L2, in contrast to MDA-MB-231 cells, which showed markedly elevated levels of both (Supplementary Fig. S4).

The presence of activating ligands such as MICA/B and ULBPs, recognized by the NKG2D receptor, typically enhances NK cell-mediated cytotoxicity. Similarly, engagement of PVR and Nectin-2 by the co-stimulatory receptor DNAM-1 can further promote NK cell activation [32]. Taken together, these ligand expression profiles suggest that both MCF-7 and MDA-MB-231 cells are immunologically accessible and capable of supporting NK cell recognition and degranulation under favourable conditions.

However, functional assays revealed that lactate markedly impairs NK cell degranulation, as evidenced by a significant reduction in CD107a surface expression. Co-culture with K562 or MDA-MB-231 cells in the presence of lactate resulted in an approximately 20% decrease in CD107a⁺ NK cells, while co-culture with MCF-7 cells induced a more substantial reduction of nearly 40%, compared to lactate-free controls (Fig. 4D).

Collectively, these findings demonstrate that lactate not only suppresses chemokine-mediated NK cell recruitment but also compromises their cytotoxic function, ultimately weakening anti-tumor immunity within the TME.

### MCT inhibition restores NK cell cytotoxic function and reduces breast cancer viability, even in a lactate-rich environment

Next, we aimed to investigate whether pharmacological inhibition of MCT transporters could attenuate the Warburg phenotype in breast cancer cells, thus restoring NK cell activity. To this end, given the appreciable expression of both MCT1 and MCT4 in breast cancer cell lines, we employed syrosingopine, a dual MCT1/MCT4 inhibitor known to block lactate and proton (H⁺) efflux [33].

As a first step, to assess the potential immunomodulatory activity of syrosingopine, we determine its sublethal concentration (IC₃₀), defined as the dose causing no more than a 30% reduction in cell proliferation in 2D cultures. To this end, MCF-7 and MDA-MB-231 cells were exposed to increasing concentrations of syrosingopine (1, 10, 25, and 50 µM) or a vehicle control (DMSO) for 48 h, yielding IC₃₀ values of 18 µM and 15 µM, respectively (Supplementary Fig. S5A).

Based on the results from 2D cultures, we selected a higher concentration range of syrosingopine (25, 50, 75, and 100 µM) for evaluation of its effect in 3D spheroid models derived from MCF-7 and MDA-MB-231 cells. The effect of the treatment on spheroid growth was assessed by measuring spheroid diameter and analyzing morphological changes. Syrosingopine inhibited spheroid growth in a dose-dependent manner in both cell lines. Additionally, the structural integrity and three-dimensional architecture of the spheroids progressively deteriorated with increasing concentrations, a phenomenon particularly evident in the MCF-7 cell line (Supplementary Fig. S5B, C). For this reason, the sublethal concentration of 25 µM was selected for subsequent 3D experiments.

Western blot analysis was performed on lysates from MCF-7 and MDA-MB-231 cells treated with syrosingopine at the IC₃₀ concentration or with vehicle control (DMSO) (Supplementary Fig. S5D, E). In line with previous reports [34], pharmacological inhibition of MCT1 and MCT4 did not lead to a significant reduction in their total protein levels, as shown by densitometric quantification, with the exception of a notable decrease in MCT4 expression in MDA-MB-231 cells (Supplementary Fig. S5E). In contrast, immunocytochemistry revealed a marked reduction in MCT1 and MCT4 staining intensity in both cell lines following syrosingopine treatment, suggesting possible changes in protein localization or surface expression rather than protein abundance (Supplementary Fig. S5F, G).

Inhibition of MCT transporters is expected to result in intracellular lactate accumulation and reduced extracellular lactate release, leading to increased acidity within the TME [33]. To assess these effects as indicators of syrosingopine activity, we measured intracellular pH in MCF-7 and MDA-MB-231 spheroids treated or untreated with syrosingopine, in the presence or absence of exogenous lactate (Supplementary Fig. S6A, B). Additionally, lactate levels in the corresponding culture supernatants were quantified (Supplementary Fig. S6C). Accordingly, a significant increase in intracellular fluorescence, indicative of decreased pH, was observed as early as 30 min after syrosingopine treatment and remained stable for up to 4 h, compared to the respective controls in both MCF-7 and MDA-MB-231 spheroids (Supplementary Fig. S6A, B). Concurrently, lactate secretion was significantly reduced in the supernatants of syrosingopine-treated MCF-7 and MDA-MB-231 spheroids compared to untreated controls (Supplementary Fig. S6C).

To determine whether metabolic reprogramming through MCT1/4 inhibition could enhance NK cell-mediated cytotoxicity against breast cancer, MCF-7 and MDA-MB-231 spheroids were treated with syrosingopine, lactate, or their combination, and subsequently co-cultured with human NK cells, either pre-exposed or not to lactate.

**Fig. 5:**
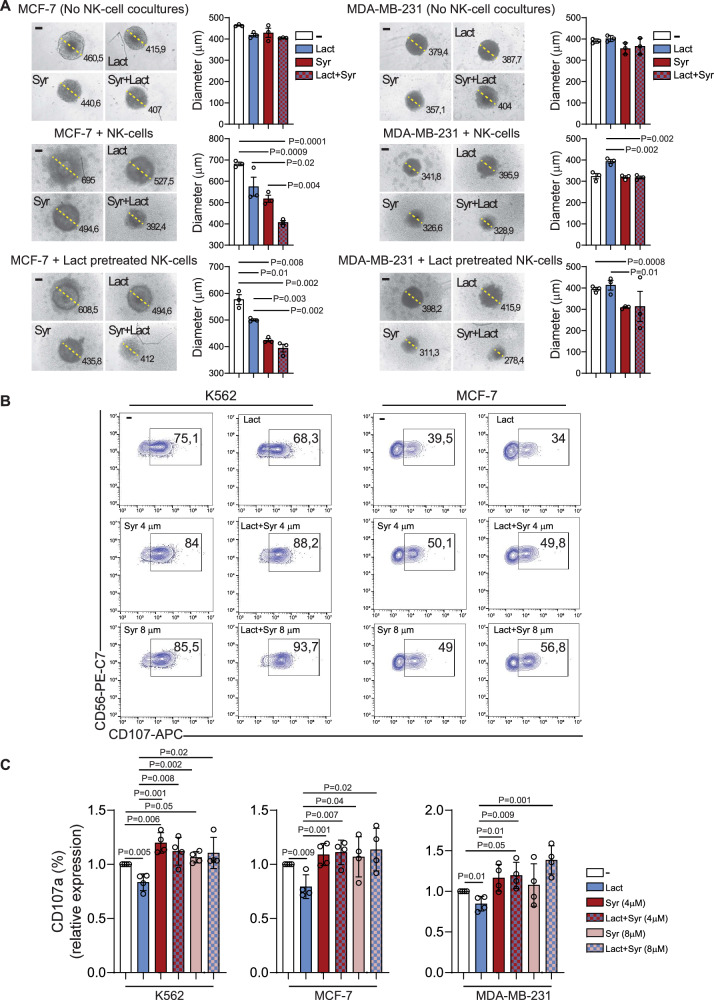
Syrosingopine reverses lactate-induced suppression of NK cell cytotoxicity and tumor control. **A** Representative images of diameter measurements in MCF-7 (left) and MDA-MB-231 (right) tumor spheroids cultured for 48 h alone (top), with NK cells (middle), or with lactate-exposed NK cells in the presence of medium alone (−), lactate (Lact), syrosingopine (Syr), or a combination of lactate and syrosingopine (Syr+Lact). On the right, bar graphs represent mean ± SEM from three independent biological experiments performed with NK cells derived from three different healthy donors (*n* = 3). Each dot represents NK cells from one donor (*n* = 3). Statistical analysis was performed using one-way ANOVA followed by Dunn’s multiple-comparison test. Exact *p*-values are reported where statistically significant; comparisons not indicated did not reach statistical significance. **B** Representative example of degranulation by human CD45^+^CD56^+^CD16^+^CD3^−^ NK cells from a healthy donor, measured as CD107a cell-surface expression following stimulation with K562 or MCF-7 target cells in the presence of medium alone (−), lactate (Lact), 4 µM (Syr 4 µM) or 8 µM syrosingopine (Syr 8 µM), or the combination of lactate with syrosingopine (Lact + Syr 4 µM or 8 µM). The percentage of CD107a⁺ NK cells is indicated. **C** Summary of NK cell degranulation using NK cells from four healthy donors (*n* = 4) after stimulation with K562, MCF-7, or MDA-MB-231 target cells under the same conditions described in (**B**). Statistical analysis was performed using one-way ANOVA followed by Dunn’s multiple comparison test. Each dot represents NK cells from one donor (*n* = 4). Exact *p*-values are reported where statistically significant; comparisons not indicated did not reach statistical significance.

In both cell lines, the absence of NK cell co-culture resulted in no significant differences among the treatment conditions, as demonstrated by comparable levels of intratumoral apoptosis at 24 h (Supplementary Fig. S7, top panels) and similar spheroid diameters at 48 h (Fig. 5A, top panels).

The addition of NK cells to tumor spheroids confirmed, in both models, a lack of significant NK cell-mediated cytotoxicity in the presence of lactate in the microenvironment, as shown by limited spheroid shrinkage (Fig. 5A, middle panels).

Notably, in NK-tumor co-culture systems, treatment with syrosingopine restored NK cell cytotoxicity in both MCF-7 and MDA-MB-231 spheroids, resulting in a significant increase in intratumoral apoptosis at 24 h (Supplementary Fig. S7, middle panels) and a reduction in spheroid size at 48 h (Fig. 5A, middle panels), compared to untreated or lactate-exposed conditions.

Importantly, this restoration of NK cell activity by syrosingopine was maintained even when NK cells were pre-exposed to additional exogenous lactate, as evidenced by persistently elevated apoptosis levels (Supplementary Fig. S7, bottom panels) and further reduction in spheroid diameter (Fig. 5A, bottom panels), relative to untreated or lactate-treated controls. This effect was particularly pronounced in MCF-7 spheroids.

To further substantiate the functional recovery of NK cells, we performed NK cell degranulation assays against K562 cells (used as a positive control), as well as MCF-7 and MDA-MB-231 breast cancer cell lines, in the presence of lactate, syrosingopine (at two concentrations), or their combination (Fig. 5B, C). For these experiments, tumor cells were pretreated with syrosingopine at the IC₃₀ concentration for 24 h. Subsequently, NK cells were added after replacing the culture medium with fresh medium containing lower concentrations of syrosingopine (4 and 8 µM), which were non-toxic to NK cells as determined in previous toxicity assays (data not shown). Flow cytometric analysis of the degranulation marker CD107a confirmed that lactate significantly suppressed NK cell activity in co-culture with all target cell lines. However, syrosingopine treatment at both 4 µM and 8 µM significantly restored CD107a surface expression, indicating a recovery of NK cell degranulation capacity. Notably, this restoration was maintained in the presence of lactate when syrosingopine was co-administered. Both concentrations of syrosingopine were effective, with the 4 µM dose producing a slightly greater effect overall. Specifically, co-culture of NK cells with K562, MCF-7, and MDA-MB-231 cells in the presence of 4 µM or 8 µM syrosingopine led to an increase in the percentage of CD107a⁺ NK cells by approximately 35% and 30%, respectively, even in the presence of exogenous lactate, compared to the lactate-only condition. When compared to the untreated condition, syrosingopine treatment resulted in an increase of about 10% for K562 and 20% for both MCF-7 and MDA-MB-231 cells.

Finally, to independently confirm that restoration of NK cell cytotoxicity was driven by inhibition of lactate transport rather than off-target effects, a complementary pharmacological approach using selective inhibitors of MCT1 and MCT4 was employed. AZD3965 (MCT1 inhibitor) and MSC-4381 (MCT4 inhibitor) were used in combination (MCTi) to repeat the same functional assays previously performed with syrosingopine in NK-tumor co-culture systems. MCF-7 and MDA-MB-231 spheroids were treated with MCTi at 0.1 µM for each compound, a concentration that did not impair tumor cell viability or NK cell fitness (data not shown).

Consistent with our previous observations, MCTi treatment alone did not affect MCF-7 and MDA-MB-231 tumor apoptosis (Supplementary Figs. S8A and S9A, left panels) or spheroid growth in the absence of NK cells (Supplementary Figs. S8B and S9B, top panels). In contrast, in the presence of NK cells, combined MCT1/4 inhibition phenocopied the effects of syrosingopine, inducing increased intratumoral apoptosis at 24 h (Supplementary Figs. S8A and S9A, middle panels), and a significant reduction in spheroid size at 48 h (Supplementary Figs. S8B and S9B, middle panels) in both MCF-7 and MDA-MB-231 models, even when NK cells were pre-exposed to exogenous lactate (Supplementary Figs. S8A, B and S9A, B, right and bottom panels respectively). Notably, MCT inhibition markedly increased CD107a surface expression on NK cells, reflecting restored degranulation capacity against both MCF-7 and MDA-MB-231 spheroids, an effect that persisted even under lactate-rich conditions (Supplementary Figs. S8C and S9C), and closely recapitulated the response to syrosingopine treatment.

Together, these data demonstrate that pharmacological inhibition of MCT1/4 restores NK cell cytotoxicity and underscore the therapeutic potential of targeting MCT1/4–mediated metabolism to enhance antitumor immunity in breast cancer.

### Integrative transcriptomic analysis reveals GPR81/HCAR1 as an orchestrator of NK cell immune invisibility in Breast Cancer

**Fig. 6:**
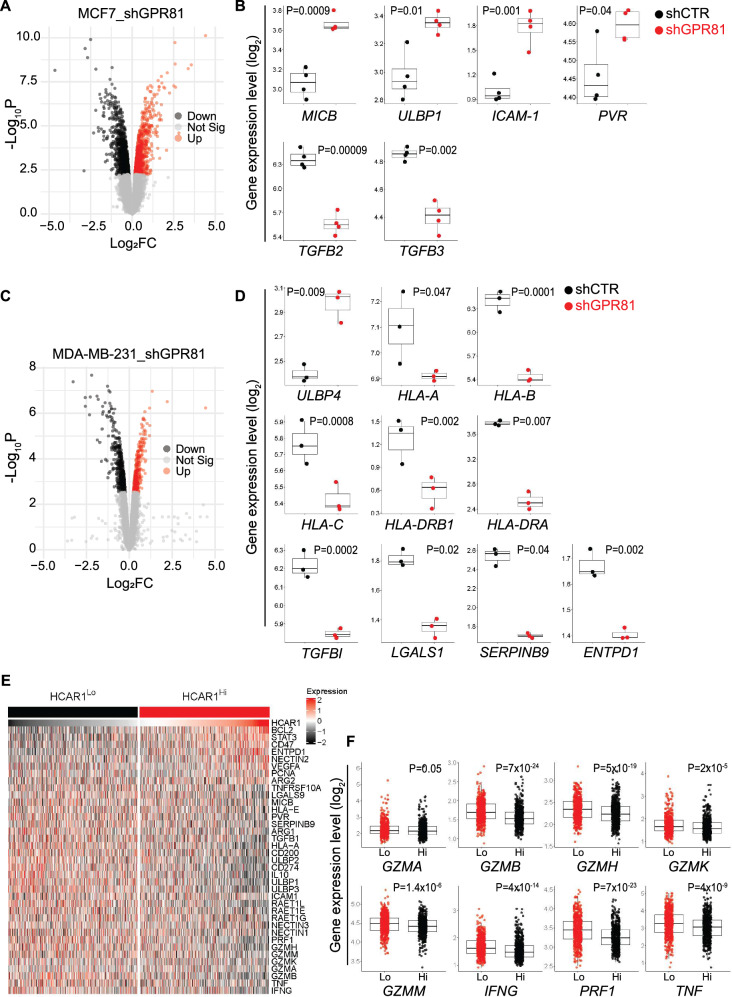
GPR81 modulates NK cell-related gene expression in breast cancer. **A** Volcano plot displaying the expression levels of GPR81-knockdown (shGPR81) MCF-7 cells compared to control (shCTR), from the RNA-seq data GSE203441 available in the GEO database. Genes significantly upregulated are shown in red (log_2_ fold change >0, *P* values < 0.05). Genes significantly downregulated are shown in black (log_2_ fold change < 0, *P* value < 0.05). **B** Box plots of expression values for the indicated NK cell related genes in shCTR and shGPR81 MCF-7 cells (GSE203441). Each dot represents one independent technical replicate (*n* = 3). **C** Volcano plot displaying the expression levels of GPR81-knockdown (shGPR81) MDA-MB-231 cells compared to control (shCTR), from the RNA-seq data GSE186211 available in the GEO database. Genes significantly upregulated are shown in red (log_2_ fold change >0, *P* values < 0.05). Genes significantly downregulated are shown in black (log_2_ fold change < 0, *P* value < 0.05). **D** Box plots of expression values for the indicated NK cell related genes in shCTR and shGPR81 MDA-MB-231 cells (GSE186211). Each dot represents one independent technical replicate (*n* = 3). **E** Unsupervised hierarchical clustering of the expression profiles of the indicated NK cell related genes in breast cancer patients with low (HCAR1lo) or high (HCAR1hi) expression of HCAR1 from the GSE115577 dataset (*n* = 882). **F** Box plots of expression values for the indicated NK cell related cytotoxic genes according to the high (Hi) and low (Lo) levels of HCAR1 mRNA (median split) in breast cancer patients from the GSE115577 dataset (*n* = 882). The dots are individual samples. In **B**, **D**, **F**, the boxes show the 25th to 75th percentile; the horizontal lines inside the box represent the median; the upper whisker extends to the largest data point, no more than 1.5 times the IQR from the box; the lower whisker extends to the smallest data point at most 1.5 times the IQR from the box. In **B**, **D**, data were analyzed by unpaired Student’s *t*-test. In **F**, *p*-values were calculated using linear modeling with Empirical Bayes moderation (limma) following a median split of HCAR1 expression. Statistically significant *p*-values are indicated.

As a final step, we sought to determine whether GPR81/HCAR1 signaling also affects NK cell–mediated immune surveillance. To this end, we performed an integrative transcriptomic analysis of breast cancer models in which GPR81 expression was genetically or transcriptionally perturbed. Public RNA-sequencing datasets from luminal MCF-7 (GSE203441) and triple-negative MDA-MB-231 (GSE186211) cells subjected to GPR81 knockdown were analyzed and compared with matched controls to identify coordinated changes in NK cell–related immune signatures (Fig. 6A–D and Supplementary Dataset S1).

In luminal MCF-7 cells, GPR81 silencing triggered a pronounced reprogramming of genes involved in NK cell recognition, adhesion, and immune regulation (Fig. 6A, B and Supplementary Dataset S1). Among the most prominent transcriptional changes was the robust upregulation of stress-induced ligands recognized by NK cells. Notably, MICB, a high-affinity ligand for the activating receptor NKG2D [35], was significantly induced. This was accompanied by increased expression of ULBP1, another NKG2D ligand [36]. In parallel, GPR81 silencing significantly increased the expression of adhesion and immune synapse-related molecules (Fig. 6B). ICAM-1, a critical ligand for LFA-1 expressed on NK cells, known to stabilize immune synapse formation [37], was strongly upregulated. Additionally, PVR, which can engage activating receptors such as DNAM-1 [38], was modestly but significantly increased. GPR81 silencing also profoundly affected immunosuppressive cytokine pathways in MCF-7 cells (Fig. 6B). Both *TGFB2* and *TGFB3* were significantly downregulated (Fig. 6B). Given the well-established role of TGF-β in suppressing NK cell activation, cytotoxicity, and receptor expression [39], this reduction is consistent with a marked alleviation of NK cell–suppressive signaling in GPR81-deficient cells.

A similar but more extensive immune reprogramming was observed in triple-negative MDA-MB-231 cells (Fig. 6C, D and Supplementary Dataset S1). In this model, GPR81 knockdown selectively upregulated NK cell stress ligands, including *ULBP4*, another ligand for the activating NK cell receptor NKG2D [40], while concurrently suppressing inhibitory signals. Indeed, multiple MHC class I and II genes (*HLA-A*, *HLA-B*, *HLA-C*, *HLA-DRA*, and *HLA-DRB1*) were significantly downregulated, consistent with reduced engagement of inhibitory NK cell receptors [41, 42]. Moreover, in addition to the downregulation of *TGFBI*, as also observed in MCF-7 cells, several key mediators of immune suppression were markedly reduced following GPR81 silencing (Fig. 6D). These included *LGALS1* (Galectin-1), a well-established inhibitor of NK- and T-cell function [43, 44], *SERPINB9*, an intracellular inhibitor of granzyme B that confers resistance to NK- and CTL-mediated cytotoxicity [45], and *ENTPD1* (CD39), a central enzyme in adenosine-driven immunosuppression [46].

Collectively, these transcriptomic analyses demonstrate that GPR81/*HCAR1* signaling exerts a broad and conserved suppressive effect on NK cell–mediated immune surveillance across distinct breast cancer subtypes, acting as a central metabolic regulator of tumor immune visibility, by simultaneously dampening NK cell activation signals and reinforcing intrinsic mechanisms of immune resistance.

To assess the clinical relevance of these experimental observations, we next analyzed NK cell-related gene modulation in a cohort of 882 breast cancer patients from the publicly available dataset GSE115577 (Fig. 6E, F and Supplementary Dataset S1). Samples were stratified according to *HCAR1* expression using a median split. Unsupervised hierarchical clustering revealed that tumors with high *HCAR1* expression exhibited a coordinated repression of genes critical for NK cell recognition and activation (Fig. 6E and Supplementary Dataset S1), including multiple NKG2D ligands (*MICB*, *ULBP1*, *ULBP2*, *ULBP3*, and *RAET1* family members), alongside reduced expression of key adhesion and immune synapse components (*ICAM1* and *PVR*). In contrast to the in vitro setting, inhibitory immune checkpoints and modulators of NK cell activity were not selectively induced but were broadly repressed, including *HLA-E*, *HLA-A*, *CD200*, *CD274* (PD-L1), *IL10*, *TGFB1*, *LGALS9*, *SERPINB9*, *ARG1*, and *TNFRSF10A* (Fig. 6E and Supplementary Dataset S1). This transcriptional profile reflects a widespread silencing of NK cell-engaging pathways, rather than compensatory upregulation of inhibitory checkpoints. Conversely, genes upregulated in *HCAR1*-high tumors were predominantly associated with tumor survival, metabolic adaptation, angiogenesis, and immune evasion through NK cell-independent mechanisms, including *BCL2*, *STAT3*, *VEGFA*, *PCNA*, *ENTPD1* (CD39), *CD47*, *ARG2*, and *NECTIN2* (Fig. 6E and Supplementary Dataset S1). Importantly, elevated *HCAR1* expression was also linked to profound suppression of NK cell cytotoxic machinery. Key effectors required for NK cell-mediated killing, including perforin (*PRF1*), multiple granzymes (*GZMA*, *GZMB*, *GZMH*, *GZMK*, and *GZMM*), and pro-inflammatory cytokines (*TNF* and *IFNG*), were markedly downregulated (Fig. 6E and Supplementary Dataset S1), indicating that *HCAR1*-high tumors both evade NK cell recognition and impair the molecular programs necessary for cytotoxic function.

Of note, this NK cell-silent phenotype (Fig. 6E, F) closely mirrored the inverse of transcriptional changes induced by GPR81 silencing in experimental models (Fig. 6A–D), highlighting a conserved and clinically relevant relationship between GPR81/*HCAR1* activity and suppression of NK cell-mediated immune surveillance. These findings suggest that, in addition to targeting MCT lactate transporters, therapeutic blockade of GPR81 could represent a complementary strategy to restore NK cell activity and enhance anti-tumor immunity in breast cancer.

## Discussion

Our study demonstrates that lactate metabolism establishes a profoundly immunosuppressive TME by impairing NK cell activation, proliferation, recruitment, and cytotoxicity in breast cancer. Through integrated transcriptomic analyses, functional in vitro assays, metabolic profiling, and 3D tumor models, we delineated a mechanistic link between lactate accumulation and NK cell dysfunction. Importantly, pharmacological inhibition of lactate transport restored NK cell effector function and reduced breast cancer cell viability, even in a lactate-rich setting, while GPR81 silencing further enhanced NK cell activity, highlighting a promising therapeutic strategy to counteract metabolic immunosuppression.

The extracellular TME is increasingly recognized as a critical determinant of cancer progression [19]. Among its constituents, lactate, a product of Warburg metabolism that is exported via MCT1/4 along with protons, acidifying the surrounding milieu [47], has historically received limited attention. Once considered merely a metabolic byproduct of hypoxia, lactate is now recognized as a key driver of metastasis [3] across multiple cancer types [48–50]. In breast cancer, our findings, consistent with recent studies [19], demonstrate that cancer cells generate substantial amounts of lactate within the TME, thereby fostering aggressive tumor traits [51]. In addition, high expression of *LDHA* and *HCAR1* was associated with poor clinical outcomes in our cohort, even after adjusting for key clinicopathological factors. These findings mirror observations in other tumors, where *LDHA* or *HCAR1* overexpression correlates with tumor progression, poor prognosis, and treatment resistance [14, 52, 53]. Our transcriptomic analysis further revealed a strong negative correlation between the expression of lactate metabolism–associated genes (*LDHA*, *HCAR1*) and NK cell–activating receptors (*NCR1*, *NCR2*, *NCR3*). Patients with high *LDHA/HCAR1* and low activating NK receptor expression exhibited the poorest recurrence-free survival, whereas those with the opposite expression profile showed the most favourable outcomes. These results are consistent with prior evidence linking elevated tumor lactate levels to increased disease aggressiveness and reduced immune infiltration across several cancer types. In melanoma, for example, *LDHA* expression negatively correlates with T-cell activation markers such as granzyme K and CD25 [14, 15]. In pancreatic cancer, the SIX1/LDHA axis has been proposed as an upstream regulator of lactate production, which exerts an inhibitory effect on NK cell function [54]. Together, these findings support the concept that lactate-driven metabolic reprogramming not only promotes tumor growth but also suppresses immune surveillance, thereby contributing to poor clinical outcomes [22, 28], although causal relationships in patients will require further validation.

To date, the impact of lactate on the immune system has been analyzed primarily in myeloid cells, which remain viable and adopt pro-tumor phenotypes in lactate-rich environments [15, 55], and in T cells, which are negatively affected by this metabolite [56]. In contrast, the effects of lactate on NK cells remain poorly investigated. The present study addresses this gap by demonstrating that lactate directly inhibits NK cell proliferation, downregulates activation markers (CD69, CD25, CD16), and reduces the expression of activating receptors (NKp30, NKp44, NKp46). Notably, this suppression does not result from overt cytotoxicity, as cell viability is largely maintained, but rather reflects functional exhaustion. In support of this, lactate exposure markedly diminishes NK cell production of IFN-γ and granzyme B, key mediators of cytotoxicity. These effects are consistent with observations in T cells infiltrating melanoma tumors, where lactate impairs their effector function [26]. In our study, analysis of NK cells from multiple donors demonstrated the expression of both monocarboxylate transporters and the lactate receptor, emphasizing the importance of this metabolic pathway for NK cell function. Similarly, activated T lymphocytes rely on efficient lactate and proton export, as intracellular accumulation disrupts metabolism and reduces ATP production [57]. As lactate transport via monocarboxylate transporters is dependent on the cytoplasmic-extracellular gradient, it has been suggested that high extracellular lactate levels could reverse the normal direction of its transportation. This would result in lactate uptake and subsequent impairment of T cell function [58, 59].

Our results further demonstrated that lactate alters the metabolic fingerprint of NK cells, decreasing cytochrome c signals and increasing lipid accumulation, indicative of disrupted mitochondrial function [60]. Bioenergetic assays confirmed reduced OCR, diminished ATP production, and impaired OxPhos efficiency, suggesting that lactate-driven mitochondrial dysfunction underlies the observed functional suppression. NK cells rely on rapid metabolic reprogramming to support cytotoxic responses [61], and our results highlight lactate as a barrier to this metabolic flexibility. These observations align with findings in liver-resident NK cells within colorectal liver metastases (CRLM), where high glycolytic activity and lactate-rich conditions induce mitochondrial stress and compromise NK-cell viability and function [16].

Beyond direct suppression of NK cell activation, we found that lactate limits NK cell recruitment to breast cancer spheroids by downregulating CXCL9 and CXCL10 secretion. This chemokine suppression impaired NK cell infiltration and reduced tumor apoptosis, despite the presence of activating ligands (MICA/B, ULBPs, PVR, Nectin-2) on tumor cells. In addition, NK cell degranulation was markedly reduced in lactate-rich environments, highlighting lactate’s multifaceted role in impairing both the trafficking and effector phases of NK cell responses. These results are consistent with other evidence supporting a role for lactate in intercellular communication within the TME. For instance, in breast cancer, lactate signalling via GPR81 reduces IFN-α production in plasmacytoid dendritic cells [62], while lactic acidosis in metastatic melanoma has been proposed to suppress cytokine secretion, including IFN-α and CXCL10 [63].

A major strength of this study is the demonstration that targeting lactate metabolism can restore NK cell function. Inhibition of MCT1/4 with syrosingopine reduced lactate efflux, acidified intracellular compartments, and reinstated NK cell degranulation and cytotoxicity, even when NK cells were pre-exposed to lactate. These effects were independently confirmed by combined MCT1/4 inhibition with AZD3965 and MSC-4381, establishing lactate transport blockade as the driving mechanism. These broad-spectrum benefits support lactate pathway blockade as a compelling therapeutic approach. Beyond transport, our data identify GPR81/*HCAR1* as a non-redundant mediator of lactate-driven immune suppression, as its ablation reprogrammed tumor cells toward an NK cell–permissive phenotype by enhancing ligands of activating receptors and attenuating immunosuppressive signaling. Together, these findings indicate that lactate suppresses NK cell immunity through both metabolic and receptor-mediated mechanisms.

Previous studies have described syrosingopine’s ability to sensitize tumors to metformin or glycolysis inhibitors [33], but its immunomodulatory role has not been previously defined. Our findings, therefore, expand the therapeutic rationale for lactate metabolism inhibitors to include NK cell–mediated immunotherapy. Supporting our results, reduced NK cell numbers, more so than T cells, have been associated with disease recurrence in CRLM, highlighting the importance of NK cell–mediated immunity in controlling tumor regrowth. Given the prominent role of lactate in CRLM, strategies aimed at reducing its production have been proposed to enhance immune surveillance in the liver and limit subsequent tumor progression, thereby potentially decreasing recurrence rates after surgery [16]. Additional evidence comes from studies on T cell–based therapies. Highly glycolytic tumor metabolism, for example, has been associated with reduced efficacy of immune checkpoint blockade [64] as well as with resistance to adoptive T-cell transfer in melanoma [65]. Accumulation of lactic acid in the TME may further impede the success of checkpoint inhibition [14], while interventions that block tumor acidification prior to immunotherapy have been shown to improve anti-tumor responses [66]. Lactate dehydrogenase levels have been proposed as a selection criterion for therapies such as ipilimumab and anti-PD-1 treatment [67]. Additionally, targeting lactate transport, for example, with anti-MCT1 antibodies, has been reported to increase the proportion of TNF-α⁺IFN-γ⁺ tumor-infiltrating lymphocytes and enhance CAR T-cell responses, supporting the concept that MCT1-dependent lactate influx constitutes a barrier that can be therapeutically overcome to improve cytotoxic lymphocyte function against solid tumors [68].

These findings provide a compelling preclinical rationale for exploring lactate inhibition as a strategy to enhance NK cell–mediated anti-tumor immunity. Translating this approach to the clinic requires careful consideration of potential off-target effects associated with both MCT inhibition, exemplified here by syrosingopine treatment, and prolonged NK-cell survival. Notably, syrosingopine has a well-established safety profile, with decades of clinical use as an anti-hypertensive and generally mild, reversible adverse effects [69]. Although MCT1/4 inhibition could theoretically disrupt physiological lactate shuttling in tissues such as muscle or brain, no acute toxicity has been observed in vivo [69]. Moreover, the metronomic dosing regimen employed here, well below the maximum tolerated levels, has been shown to be immunomodulatory and is associated with a low incidence of systemic side effects [70]. Regarding long-term NK-cell viability, NK cells are generally safe due to their limited lifespan and restrained cytokine release; however, prolonged activation could, in principle, trigger non-specific inflammation or off-tumor effects [71]. Clinical experience, including CAR-NK studies, suggests these risks are comparatively low, underscoring the importance of balancing durable NK-cell functionality with controlled activation in future translational applications [71].

Collectively, our findings establish lactate metabolism as a central regulator of breast cancer immune evasion, suppressing NK-cell activation, metabolism, recruitment, and cytotoxicity through both direct and indirect mechanisms. From a clinical perspective, the inverse relationship between lactate pathway activity and NK-cell receptor expression is independently associated with disease recurrence after multivariable adjustment, highlighting its relevance as a biologically meaningful immune–metabolic signature. From a therapeutic standpoint, pharmacological blockade of lactate transporters and receptor emerges as a promising approach to recondition the TME and potentiate NK cell-based therapies, including adoptive NK-cell transfer, cytokine stimulation, and immune checkpoint blockade.

Although our functional assays were performed exclusively in vitro, these results provide a compelling rationale for future validation using patient-derived samples from breast cancer and other lactate-producing tumors.

In conclusion, by demonstrating that lactate blockade restores NK-cell activity in lactate-rich conditions, our study positions metabolic intervention as a viable approach to counteract immunosuppression in the TME and supports further investigation in translational and clinical settings.

## Materials and methods

### In silico analysis

The correlation between the lactate metabolic pathway and NK cell functionality was examined using gene expression data from the publicly available breast cancer dataset GSE115577 (*n* = 882 invasive breast cancer patients), accessed via the R2 Genomics Analysis and Visualization Platform (http://r2.amc.nl). Only correlations with *r* ≥ 0.3 and *p* < 0.05 were considered significant. Event-free survival curves for groups stratified by different criteria were generated using the Kaplan–Meier method implemented in the *survival* R package and compared using the log-rank test [72]. Statistical significance was defined as *p* < 0.05.

#### Multivariate analyses

To further assess the independent and joint prognostic impact of lactate metabolism–related genes and NK cell activation markers, multivariable survival analyses were performed.

The joint associations between *HCAR1* or *LDHA* expression and *NCR1, NCR2*, and *NCR3* were evaluated using multivariable survival models adjusted for age at diagnosis, tumor grade, HER2 status, ER status, and tumor size. Multivariable Cox proportional hazards models were fitted when the proportional hazards (PH) assumption was satisfied, as assessed using Schoenfeld residuals. Results from Cox models are reported as hazard ratios (HRs) with 95% confidence intervals (CIs). When the PH assumption was violated, restricted mean survival time (RMST) analyses were performed using pseudo-values and generalized estimating equations. RMST was estimated up to a prespecified truncation time (τ), chosen as the minimum of the largest observed event times across comparison groups to ensure valid estimation. RMST results are reported as differences in RMST (ΔRMST, in years) with 95% CIs, representing the average gain or loss in recurrence-free survival time relative to the reference group. For RMST analyses, a positive ΔRMST indicates longer recurrence-free survival (RFS) compared with the reference group, whereas a negative ΔRMST indicates shorter RFS. For Cox models, HRs greater than 1 indicate increased risk of recurrence, and HRs less than 1 indicate reduced risk.

#### GPR81 signaling analysis

Differential gene expression analysis was performed using the limma package in R. For dataset GSE203441 (related to MCF-7 cells), CPM-normalized count data were filtered to retain only lactate-treated samples, which were then stratified into control (pLKO) and GPR81 knockdown (shGPR81) groups. For dataset GSE186211 (related to MDA-MB-231 cells), FPKM values were log2-transformed after adding a pseudocount of 1, and genes with expression greater than 1 in at least 25% of samples were retained for analysis.

For both datasets, linear models were fitted using the lmFit function with a design matrix encoding the experimental groups. Differential expression between shGPR81 and control conditions was assessed using empirical Bayes moderated t-statistics implemented in the eBayes function. Genes were considered differentially expressed at an adjusted *p*-value < 0.05. For GSE203441, Ensembl gene identifiers were mapped to gene symbols using the org.Hs.eg.db annotation package.

Results were visualized using volcano plots generated with ggplot2, and sample-level relationships were assessed using multidimensional scaling (MDS) plots. Expression levels of selected genes of interest were compared between conditions using two-sample t-tests and visualized as boxplots.

For breast cancer patients, gene expression data from the GSE115577 dataset were obtained and merged with probe-level gene annotations to map probes to gene symbols. Samples corresponding to the NHS cohort were extracted based on clinical metadata. For genes represented by multiple probes, expression values were averaged using the avereps function from limma.

Samples were stratified into HCAR1-high and HCAR1-low groups based on whether HCAR1 expression was above or below the median expression value across all samples. A predefined set of genes of interest was selected for visualization. Expression values were row-scaled (z-score normalized) across samples, and values were clipped to a range of ±4 for visualization purposes.

Heatmaps were generated using the ComplexHeatmap package in R.

### Cell lines and reagents

Human breast cancer cell lines MDA-MB-231 and MCF-7 (ATCC) were cultured in Dulbecco’s Modified Eagle Medium (DMEM; Thermo Fisher Scientific). The human erythroleukemia cell line K562 (ATCC), which served as a control target in the NK cell degranulation assays, was cultured in RPMI-1640 medium. All media were supplemented with 10% fetal bovine serum (FBS, Thermo Fisher Scientific), 2 mM L-glutamine, 100 µg/mL penicillin, and 50 µg/mL streptomycin (EuroClone S.p.A.). Cells were maintained at 37 °C in a humidified atmosphere containing 5% CO₂ and routinely tested for mycoplasma contamination. Sodium L-lactate (L7022), Syrosingopine (SML1908), MSC-4381 (SML3470), and AZD3965 (SML4345) were from Sigma-Aldrich.

### Generation of 3D human breast tumor spheroids

Tumor spheroids were generated by seeding 100 µL/well of MCF-7 or MDA-MB-231 cells (4000 cells/well) in 96-well round-bottom ultra-low attachment (ULA) plates (Corning). Plates were centrifuged at 2000 rpm for 2 min at room temperature to promote aggregation, then incubated at 37 °C with 5% CO₂ and 95% humidity for 48 h to allow spheroid formation.

### Healthy donors and breast cancer patients

This study included voluntary blood donors recruited at the transfusion centers of Bambino Gesù Children’s Hospital, IRCCS (Rome, Italy), and Ospedale Policlinico San Martino, IRCCS (Genoa, Italy), whose samples were used for NK cell isolation. In addition, plasma samples from 13 patients with breast cancer (7 with luminal subtype and 6 with triple-negative breast cancer, TNBC) were collected prior to any chemotherapy from Ospedale Policlinico San Martino, IRCCS (Genoa, Italy) and used to assess circulating lactate levels. All biological samples were obtained after written informed consent, in accordance with the Declaration of Helsinki, and with approval from the respective local Ethics Committees (Prot. n. 127/2022-DB and n. 39/2012).

### Primary NK cell isolation and expansion

NK cells were isolated directly from whole blood using the RosetteSep™ Human NK Cell Enrichment Kit (STEMCELL Technologies), following the manufacturer’s instructions (50 µL enrichment cocktail was mixed with 1 mL of whole blood, and incubated for 20 min at room temperature). Mononuclear cells were separated by density centrifugation with Ficoll-Paque Plus (GE Healthcare), and NK cells (>90% purity) were resuspended in NK MACS medium (Miltenyi Biotec) supplemented with NK MACS supplement, AB human serum, and 500 IU/mL recombinant human IL-2 (PeproTech). Cells were cultured at 37 °C in a humidified 5% CO₂ incubator, split every 3 days, and used for experiments within 20 days post-isolation.

### Western blotting

Western blot analysis was performed on MDA-MB-231 and MCF-7 cell lines, as well as on human NK cells isolated from healthy donors. Whole-cell lysates were prepared in RIPA buffer (25 mM Tris–HCl, pH 8.8; 150 mM NaCl; 5 mM EDTA; 1% Triton X-100; 1% sodium deoxycholate; and 0.1% SDS) and clarified by high-speed centrifugation. Protein concentrations were determined using the bicinchoninic acid (BCA) Protein Assay Kit (Thermo Fisher Scientific), and lysates were normalized accordingly. Equal amounts of protein (30 µg per lane) were loaded onto 10% SDS-PAGE gels and transferred to nitrocellulose membranes at 300 mA for 2 h in transfer buffer (25 mM Tris, 192 mM glycine, 10% methanol). Membranes were blocked with 5% non-fat milk in TBS containing 0.5% Tween-20 (TBST) and incubated overnight at 4 °C with primary antibodies (anti-MCT1; anti-MCT4; anti-GPR81; or anti-Vinculin, detailed in Supplementary Table 2). After washing, membranes were incubated with HRP-conjugated secondary antibodies for 1 h at room temperature (Supplementary Table 2). Signal detection was performed using the Western Lightning ECL Pro chemiluminescence kit (PerkinElmer), and images were acquired with a ChemiDoc imaging system (Bio-Rad). Band intensities were quantified by densitometry using ImageJ software (NIH, Bethesda, MD), and protein levels were normalized to Vinculin. Full-length Western blots and high-resolution images are shown in Supplementary Figs. S10 and S11.

### Immunocytochemistry

Immunocytochemistry was performed on MDA-MB-231 and MCF-7 cell lines seeded in 8-chamber polystyrene vessels containing tissue culture-treated glass slides (Corning Falcon), as well as on NK cells deposited onto microscopy slides using cytospin. Cells were fixed with 4% paraformaldehyde, washed with phosphate-buffered saline (PBS), and permeabilized with 0.1% Triton X-100. After blocking with 5% bovine serum albumin (BSA) for 30 min at room temperature, cells were incubated overnight at 4 °C with anti-human MCT1 and anti-human MCT4 primary antibodies (Supplementary Table 2). Primary antibody binding was detected using the Ultra-Tek HRP anti-polyvalent system (AFN600, ScyTek) and visualized with the DAB Chromogen System (ACH500, ScyTek), in which horseradish peroxidase–conjugated polymers catalyzed the color reaction. Images were acquired using a Leica DMLB microscope and analyzed with the IHC Profiler plugin for ImageJ (NIH).

### Quantification of lactate secretion

To assess L-lactate production, plasma of breast cancer patients and supernatants from breast cancer spheroids were collected and analyzed using a colorimetric Lactate Assay Kit (Cell Biolabs), according to the manufacturer’s instructions. Samples were centrifuged at 10,000 rpm to remove insoluble particles and subsequently diluted as needed. Each sample was analyzed in duplicate. Optical density (OD) was measured at 570 nm using a microplate reader, and lactate concentrations were determined by comparison to a standard curve.

### NK cell viability assay

Human NK cells were seeded in 96-well plates at a density of 2 × 10⁵ cells/well and exposed to increasing concentrations of sodium L-lactate (10, 20, 30, 40, 50, and 60 mM) or left untreated. Viability was assessed at 24 h and 48 h using the trypan blue exclusion (TBE) method, which distinguishes live from dead cells based on their differential uptake of the dye reagent. An equal volume of 0.4% (w/v) trypan blue (BioWhittaker®, Lonza Bioscience) was mixed with the cell suspension at a 1:2 cell-to-reagent ratio, and viable and non-viable cells were counted using a Bürker chamber.

### Flow-cytometry analysis

All antibodies were purchased from BD Biosciences, eBioscience, Biolegend, or R&D Systems (listed in Supplementary Table 2).

For the apoptosis assay, the PE Annexin-V/7-AAD Detection Kit (BD Pharmingen) was used following the manufacturer’s instructions. NK cells were seeded in 96-well U-bottom plates at a density of 1 × 10⁵ cells/100 µL and exposed, or not, to 60 mM sodium L-lactate for 24 h or 48 h. Cells were then collected, resuspended in 100 µL of 1× Annexin V Binding Buffer (0.1 M HEPES/NaOH, pH 7.4, 1.4 M NaCl, 25 mM CaCl₂), and incubated with 2 µL of PE–Annexin V and 2 µL of 7-AAD for 20 min at room temperature in the dark.

For surface staining, cells were incubated with fluorochrome-conjugated antibodies in PBS supplemented with 1% FBS for 20 min at 4 °C. Viability was assessed using Fixable Live/Dead Zombie dye (BioLegend). For intracellular staining, cells were first stained for surface markers, then permeabilized with Permeabilization Buffer (Invitrogen) and fixed with FoxP3/Transcription Factor Fixation/Permeabilization Solution (Invitrogen), followed by staining with anti-IFN-γ and anti-granzyme B antibodies.

Flow cytometry data were acquired on a CytoFLEX cytometer (Beckman Coulter) and analyzed using FlowJo software, version 10.0.8r1 (Tree Star, Ashland, OR, USA) or CytExpert software, version 2.5.

### Real-time monitoring of extracellular pH

To verify the pH of sodium L-lactate-containing media compared to that of lactate-free controls, under physiological osmolarity, extracellular pH was monitored in real time using an optical, contactless pH sensing system integrated into a 96-well plate (PyroScience, Germany). To this end, NK cells were cultured in control medium, medium supplemented with 60 mM sodium L-lactate, or HCl-adjusted medium (pH ~6.4) to mimic in vivo TME acidification [22]. The system enabled measurements across a defined pH range (5.5–8.5) under standard incubator conditions. pH-sensitive sensor spots (3 mm in diameter and 50 μm thick) were punched from a photosensitive film (PyroScience, Germany) and sealed to the center of each well using silicone adhesive. Measurements were acquired using a USB-powered FireSting®-PRO fiber-optic meter (PyroScience, Germany) equipped with three fiber-optic cables and an external Pt100 temperature probe for automatic temperature compensation.

### Measurement of intracellular pH

Intracellular pH (pHi) was measured using the pH-sensitive fluorescent dye pHrodo™ Red AM (Molecular Probes), following the manufacturer’s protocol. Following treatment (for NK cells with control medium, sodium L-lactate, or acidified medium; for tumor spheroids from MDA-MB-231 and MCF-7 cell lines with DMSO, sodium L-lactate, syrosingopine, and sodium L-lactate plus syrosingopine in the co-culture systems described below), cells were washed with HBSS containing 20 mM HEPES and incubated with pHrodo™ Red AM ester for 30 min at 37 °C. Fluorescence, which correlates with intracellular pH (calibrated using standards from Thermo Fisher Scientific, cat. no. P35379), was imaged using an Olympus IX50 microscope and quantified with ImageJ software and acquired by flow cytometry.

### Raman imaging

NK cells, both exposed and unexposed to lactate, were deposited on CaF₂ slides and measured in back scattering geometry after drop-drying. Several cells from each of the two groups were spectrally analysed using a Thermo Fisher Scientific DXR2xi Raman imaging microscope equipped with a 532 nm excitation laser and a 50x objective. Each spectrum acquired on a single cell was collected at 6 mW laser power with 1000 accumulations, each lasting 1 s. The average spectra calculated on each group of cells were compared after smoothing (Savitzky-Golay filter with a 5-point window), fluorescence background subtraction (polynomial of order 3), and baseline correction. No further normalization was applied, but rather the ratios between specific peaks were calculated as quantifiers of the balance between respiratory activity and lipid metabolism.

### Metabolic assays

Oxidative phosphorylation (OxPhos) function was assessed by measuring the oxygen consumption rate (OCR) and the activity of the F_o_F_1_-ATP synthase, as previously performed [27]. Unless otherwise indicated, reagents were purchased from Merck.

#### Oxygen consumption rate (OCR) evaluation

OCR was measured using an amperometric oxygen electrode (Unisense Microrespiration system; Unisense A/S, Aarhus, Denmark) in a sealed respiration chamber containing phosphate-buffered saline (PBS; 1.7 mL final volume). For each measurement, 1 × 10^5^ cells were resuspended in PBS supplemented with digitonin (0.03 mg/mL) to permeabilize the plasma membrane and allow access of exogenous respiratory substrates to mitochondria. After 1 min of incubation with digitonin, cells were transferred into the sealed chamber, and basal respiration was recorded.

Mitochondrial respiration was then stimulated using one of the following substrate conditions: (i) pyruvate (10 mM) plus malate (5 mM) to support a complex I–linked respiratory pathway (complexes I, III, and IV), or (ii) succinate (20 mM) to support a complex II–linked respiratory pathway (complexes II, III, and IV). In both cases, substrate addition was immediately followed by the addition of ADP (0.1 mM) to quantify ADP-stimulated respiration associated with ATP synthesis.

#### F_o_F_1_-ATP synthase activity and ATP production

F_o_F_1_-ATP synthase–dependent ATP production was measured in a suspension of 1 × 10^5^ cells in PBS supplemented with ouabain (0.6 mM) and di(adenosine)-5′-pentaphosphate (Ap_5_A; 0.25 mM) to inhibit Na^+^/K^+^-ATPase and adenylate kinase activity, respectively, thereby minimizing non-mitochondrial ATP consumption and ATP interconversion that could interfere with detection. Cells were incubated with these inhibitors for 10 min and then stimulated with the same respiratory substrates used for OCR measurements (pyruvate 10 mM plus malate 5 mM, or succinate 20 mM) together with ADP (0.1 mM).

ATP production was quantified using a luciferin/luciferase-based chemiluminescence assay (ATP Bioluminescence Assay Kit CLS II, #11699695001; Roche) on a GloMax® 20/20 luminometer (Promega). Luminescence readings were collected every 30 s over a 2 min interval.

#### Calculation and interpretation of the P/O ratio

Because both ADP-stimulated OCR and ATP production were obtained under matched substrate conditions, the P/O ratio (ATP produced per oxygen atom consumed) was calculated as an index of OxPhos coupling efficiency. In practice, ATP production rates derived from the luminescence assay were normalized to the corresponding ADP-stimulated oxygen consumption rates measured by the electrode. Under conditions of tight OxPhos coupling, where oxygen consumption is fully linked to ATP synthesis, stoichiometric studies predict P/O values of approximately 2.5 for complex I–linked respiration (pyruvate/malate) and approximately 1.5 for complex II–linked respiration (succinate). P/O values below these reference stoichiometries indicate that a fraction of oxygen consumption is not coupled to ATP production (e.g., due to proton leak/uncoupling and/or other non-phosphorylating oxygen-consuming reactions, potentially including ROS-generating processes).

### Microfluidic devices

The immunomodulatory effects of lactate were assessed using microfluidic devices fabricated from polydimethylsiloxane (PDMS), a biocompatible silicone elastomer, via standard replica moulding techniques [73]. Prior to cell loading, the devices were sterilised under UV light for 30 min. Tumor spheroids derived from MCF-7 and MDA-MB-231 cell lines were resuspended in Matrigel (2 mg/mL; BD Biosciences) and supplemented with or without exogenous lactate. Both lactate-treated and untreated spheroids (1 × 10⁴ cells in 3 μL) were placed in the side chambers of the microfluidic devices, separated from the central chamber by microchannels, and incubated at 37 °C for 30 min to allow Matrigel polymerisation.

Next, NK cells (1 × 10⁶) isolated from different healthy donors were labelled with CellTracker™ Red (Invitrogen), suspended in complete RPMI medium, and introduced into the central chamber. The microchannel architecture enabled directional migration of NK cells toward the spheroids while preventing spheroid displacement into the central chamber. For NK cell migration analysis, brightfield and fluorescence images were acquired using an OLYMPUS IX50 microscope 48 h after loading, and the red-labelled cells entered in the left or right side chambers were quantified using ImageJ v1.54j (http://imagej.net/ij), as previously performed [29, 30].

In addition, to assess NK cell-induced apoptosis within tumor spheroids under lactate-poor or lactate-rich conditions, CellEvent™ Caspase-3/7 Green detection reagent (Invitrogen) was added to the side chambers of the microfluidic devices. After 48 h, fluorescence intensity was measured using ImageJ, and apoptotic activity was quantified.

### Determination of CXCL9 and CXCL10 chemokines

Quantification of CXCL9 and CXCL10 levels was performed using DuoSet ELISA kits (DY266 and DY392, respectively; R&D Systems) according to the manufacturer’s instructions. Ninety-six–well polystyrene plates were coated overnight at room temperature with 100 μL per well of 800 ng/mL anti-CXCL9 or 2 μg/mL anti-CXCL10 antibodies in PBS. The following day, plates were washed three times with 400 μL of wash buffer and blocked with 1% BSA for 1 h at room temperature. After an additional three washes, samples and standards were added and incubated for 2 h. Plates were then washed three times and incubated with 100 μL per well of 200 ng/mL biotinylated anti-CXCL9 or 100 ng/mL biotinylated anti-CXCL10 for 2 h at room temperature. Following another series of three washes, streptavidin-HRP was added and incubated for 20 min. Detection was carried out using tetramethylbenzidine (Sigma-Aldrich) and H₂O₂ as the substrate. Absorbance was measured at 450 nm using a microplate reader (AMR-100, Dutscher).

### Degranulation assay

NK cells were co-cultured with K562, MCF-7, or MDA-MB-231 cell lines at a 1:1 ratio for 3 h in complete medium and in the presence of anti-CD107a antibody at a 1:100 dilution. During the final 2 h of co-culture, GolgiStop (BD Biosciences) was added at a 1:500 dilution to inhibit protein transport. Following incubation, cells were stained with anti-CD45, anti-CD56, anti-CD16, and anti-CD3 antibodies (see Supplementary Table 2). CD107a expression was then assessed by flow cytometry within the CD45⁺CD3⁻CD56⁺CD16⁺ NK cell population.

### 2D and 3D proliferation assays following Syrosingopine treatment

For 2D cultures, the sulforhodamine B (SRB) assay was used to assess the survival of MDA-MB-231 and MCF-7 cell lines following treatment with syrosingopine (Sigma-Aldrich). Cells were seeded in flat-bottom 96-well plates at a density of 5 × 10³ cells per well and allowed to adhere for 24 h. Subsequently, cultures were treated with increasing concentrations of the MCT drug inhibitor syrosingopine (1 µM, 10 µM, 25 µM, or 50 µM) or with 0.1% DMSO as a vehicle control in complete medium. To fix the cells, cold 10% trichloroacetic acid (TCA) (cat. no. T0699, Merck-Sigma Aldrich) was added to each well and incubated for 1 h at 4 °C. Wells were then rinsed with distilled water, air-dried, and stained for 30 min with 0.4% (w/v) SRB solution (cat. no. S1402, Merck-Sigma Aldrich) in 1% acetic acid. After four washes with 1% acetic acid, plates were air-dried, and the protein-bound dye was solubilized by adding 100 µL of 10 mM Tris buffer (pH 10) to each well. Absorbance was measured at 540 nm using a spectrophotometric plate reader. Cell survival was expressed as a percentage relative to the DMSO-treated control, based on normalized OD values.

For 3D cultures, the effect of syrosingopine was evaluated by treating tumor spheroids derived from MCF-7 and MDA-MB-231 cell lines with 25, 50, 75, or 100 µM syrosingopine, or with DMSO as a vehicle control, in complete medium. Spheroid imaging was performed after 24 and 48 h of incubation using an Olympus IX50 inverted optical microscope. Spheroid diameters were measured using Capture 3.0 software.

### Syrosingopine treatment and co-culture experiments

MCF-7 and MDA-MB-231 tumor spheroids were exposed for 48 h to the following conditions: (i) DMSO (vehicle control), (ii) 60 mM sodium L-lactate, (iii) 25 µM syrosingopine, and (iv) 60 mM sodium L-lactate + 25 µM syrosingopine. After treatment, the medium was removed, and 4 × 10⁵ NK cells were added to each condition. Treatments were then renewed using 8 µM syrosingopine, a non-toxic dose for immune cells.

The immunomodulatory effects of syrosingopine on NK cell-mediated cytotoxicity were assessed at two time points: at 24 h using CellEvent™ Caspase-3/7 Green detection reagent (Invitrogen), and at 48 h by measuring tumor spheroid diameter (both methods described above).

Additionally, NK cell degranulation assays (as described above) were performed using two concentrations of syrosingopine (4 µM and 8 µM) to further evaluate its impact on NK cell function.

### Co-culture of MCF-7 and MDA-MB-231 tumor spheroids with human NK cells under alternative dual MCT1/4 inhibition

To validate the syrosingopine-mediated modulation of NK cell cytotoxicity, co-culture experiments were established using alternative inhibitors targeting MCT1 and MCT4 (MCTi). MCF-7 and MDA-MB-231 tumor spheroids were generated from 2D cultures. On day 2 post-seeding, spheroids were treated with the combination of MCT inhibitors, while NK cells were cultured in the presence or absence of 60 mM sodium L-lactate. The following day, co-culture conditions were initiated as follows: (i) Tumor spheroids treated with DMSO (vehicle control) plus NK cells; (ii) Tumor spheroids treated with 0.1 µM MCT1i (AZD3965) combined with 0.1 µM MCT4i (MSC-4381) plus NK cells; (iii) Tumor spheroids treated with DMSO plus lactate-pretreated NK cells; (iv) Tumor spheroids treated with 0.1 µM MCT1i combined with 0.1 µM MCT4i plus lactate-pretreated NK cells. A control condition with tumor spheroids treated with DMSO or MCTi in the absence of NK cells was also included. Tumor spheroid apoptosis and diameter were assessed using CellEvent™ Caspase-3/7 Green detection reagent (Invitrogen) after 24 h and by microscopy acquisition after 48 h, respectively (methods described above). Finally, NK cell degranulation assays were performed after 48 h, under the corresponding treatment conditions.

### Statistical analysis

Statistical analyses were performed using GraphPad Prism version 8.0.2. The specific statistical tests used are detailed in the figure legends. Unless otherwise stated, all data are representative of at least three independent biological experiments, each with three independent technical replicates. Error bars indicate the standard error of the mean (SEM), based on triplicate experimental conditions. A *p*-value ≤ 0.05 was considered statistically significant.

## Supplementary information

Supplementary Figures and Figure Legends

Supplementary Tables

Supplementary Dataset 1

## Data Availability

The authors declare that all data supporting the findings of this study are available within the paper and its supplementary information files. The bioinformatic investigation was performed by querying gene expression cancer datasets from GEO GSE115577, GSE203441, and GSE186211. Any other relevant data and code are available from the corresponding author upon reasonable request.
