# Supplementary material for: Lactate-mediated NK cell dysfunction as a prognostic marker and therapeutic target in breast cancer
Source: Cell Death Discov. 2026 Mar 27;12:200. doi: 10.1038/s41420-026-03063-5 (PMC13149829; doi:10.1038/s41420-026-03063-5)
Supplement: Supplementary file 1 — Supplementary Figures and Figure Legends [file 41420_2026_3063_MOESM1_ESM.pdf]

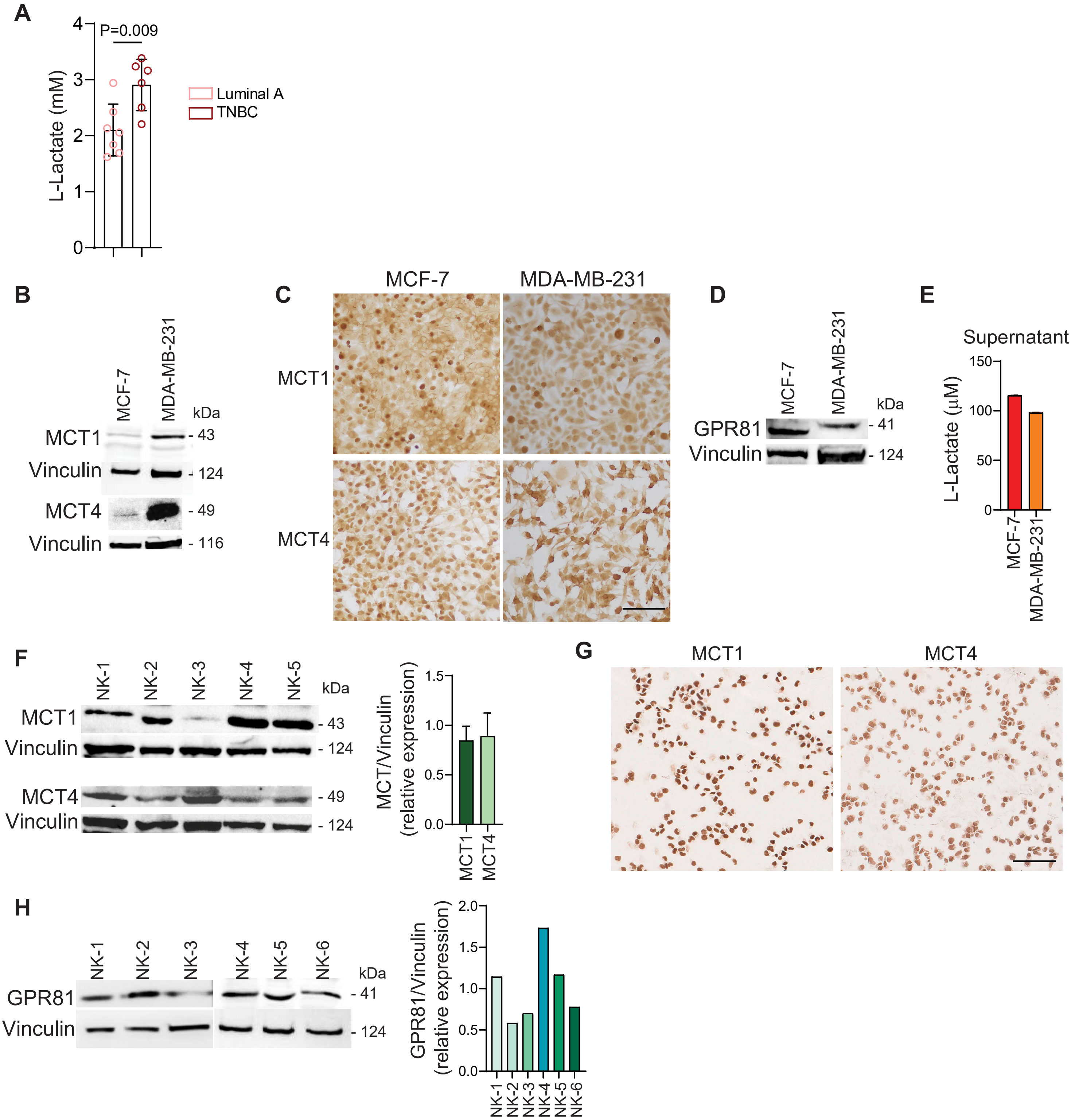

Supplementary figure S1 related to Figure 2. Lactate transporter and receptor profiles in Breast Cancer Cell lines and healthy donor-derived NK Cells. A, Measurement of plasma L-lactate levels in breast cancer patients stratified by luminal (n=7) and TNBC (n=6) subtypes. Data are presented as mean ± SEM. Each dot represents one breast cancer patient. Statistical significance was assessed using a two-tailed Student's t-test, and exact *p*-value is reported in the corresponding plot. **B**, Representative immunoblot analysis of MCT1 (top) and MCT4 (bottom) expression in MCF-7 and MDA-MB-231 cell lines. **C**, Representative immunocytochemistry images of MCT1 (top) and MCT4 (bottom) in MCF-7 and MDA-MB-231 cell lines. Positive cells are stained in brown. Original magnification ×20; scale bar, 30 μm. **D**, Representative immunoblot analysis of GPR81 expression in MCF-7 and MDA-MB-231 cell lines. **E**, Measurement of L-lactate production in supernatants of MCF-7 and MDA-MB-231 cell lines, from two independent biological experiments (n=2). Data are presented as mean ± SEM. Statistical analysis was not performed due to the low sample size. **F**, Representative immunoblot analysis of MCT1 and MCT4 expression in NK cells derived from five different healthy donors (n=5). Average densitometric quantification of NK cells from five independent donors, normalized to vinculin is shown on the right.Data are presented as a summary of independent donors; no statistical comparisons were performed. **G**, Representative immunocytochemistry images of MCT1 and MCT4 in NK cells from one healthy donor. Positive cells are stained brown. Original magnification ×20; scale bar, 30 μm. **H**, Representative immunoblot analysis of GPR81 expression in NK cells from six different healthy donors (n=6). Densitometric quantification normalized to vinculin is shown on the right. Data are presented as a summary of independent donors; no statistical comparisons were performed.

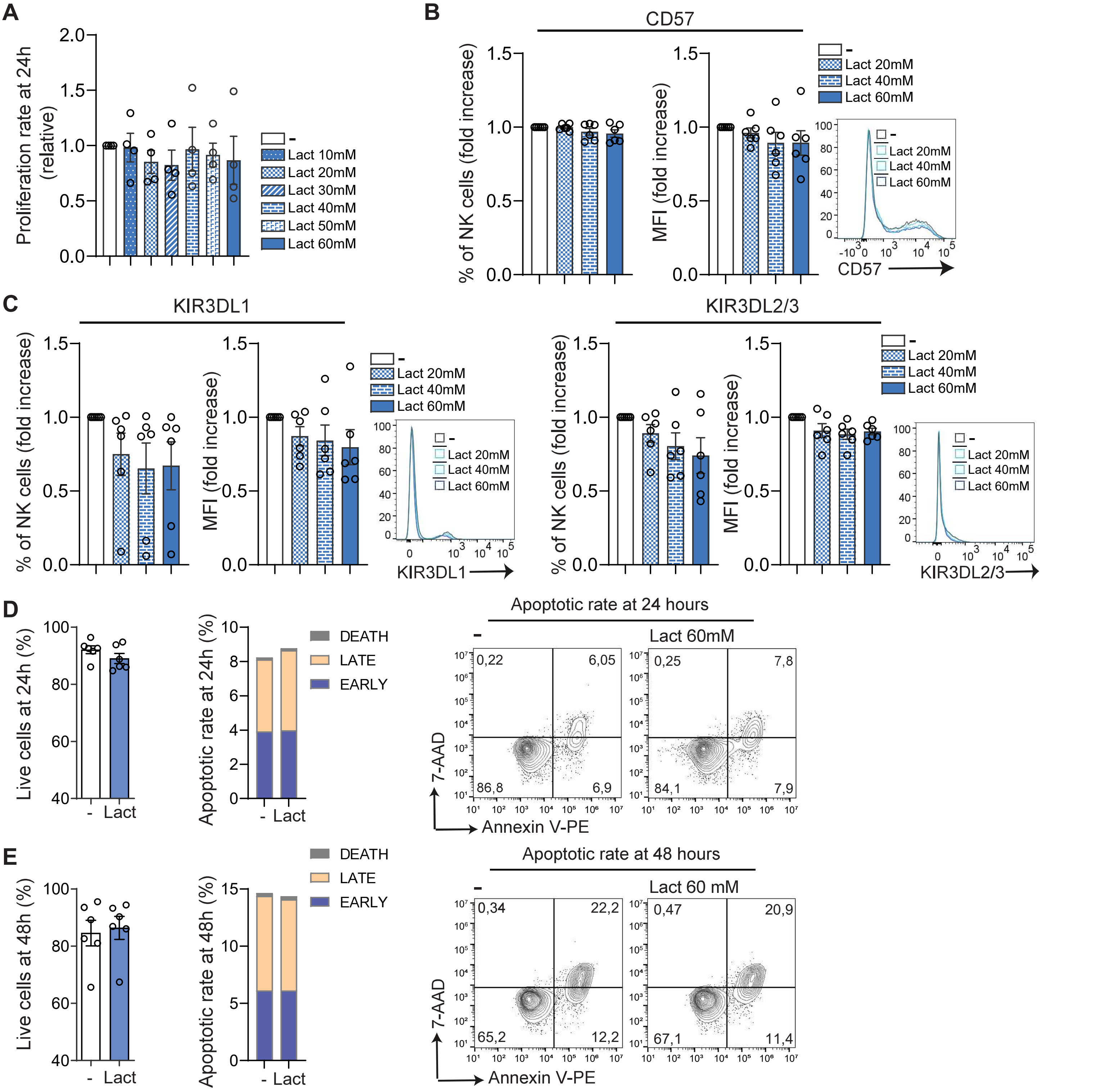

Supplementary figure S2 related to Figure 2. Effects of increasing lactate concentration on NK cell viability, phenotype, and function. A, NK cell proliferation rate after 24 h incubation with the indicated concentrations of sodium L-lactate (Lact) or without lactate (−). Data represent mean ± SEM from independent biological replicates performed with NK cells derived from four independent donors. Each dot represents one donor (n=4). Statistical significance was determined using one-way ANOVA followed by Dunn's multiple-comparison test. Exact *p*-values are reported in the plot. **B-C**, Expression of (B) CD57 (n=6) and (C) inhibitory receptors KIR3DL1 (n=6) and KIR3DL2 (n=6) on NK cells cultured with the indicated concentrations of lactate (Lact) or without lactate (−). Data are shown as both percentage of positive cells (left for B, C, D, F) and MFI fold change (right) relative to untreated controls. Representative flow-cytometry histograms depicting MFI shifts for each marker are also shown. Data represent mean ± SEM from independent biological replicates using NK cells from six healthy donors (n=6); each dot represents one donor. Statistical significance was assessed using one-way ANOVA followed by Dunn's multiple-comparison test; exact *p*-values are reported in the corresponding plots. **D**,**E**, Annexin V-PE/7-AAD staining assay. Left, percentages of live and apoptotic NK cells from independent experiments using NK cells from different healthy donors (n=6) after 24 h (D) and 48 h (E) of culture with 60 mM lactate (Lact) or without lactate (−). Statistical significance was assessed using a two-tailed Student's t-test; comparisons in D and E were not statistically significant ($p > 0.05$) and are therefore not indicated in the plots. Right, representative flow cytometry plots showing the percentages of NK cells in early apoptosis (Annexin V^+ 7-AAD^-), late apoptosis (Annexin V^+ 7-AAD^+), and dead cells (Annexin V^- 7-AAD^+) after 24 hours (D) and 48 hours (E) with 60mM lactate (Lact) or without (−) lactate.

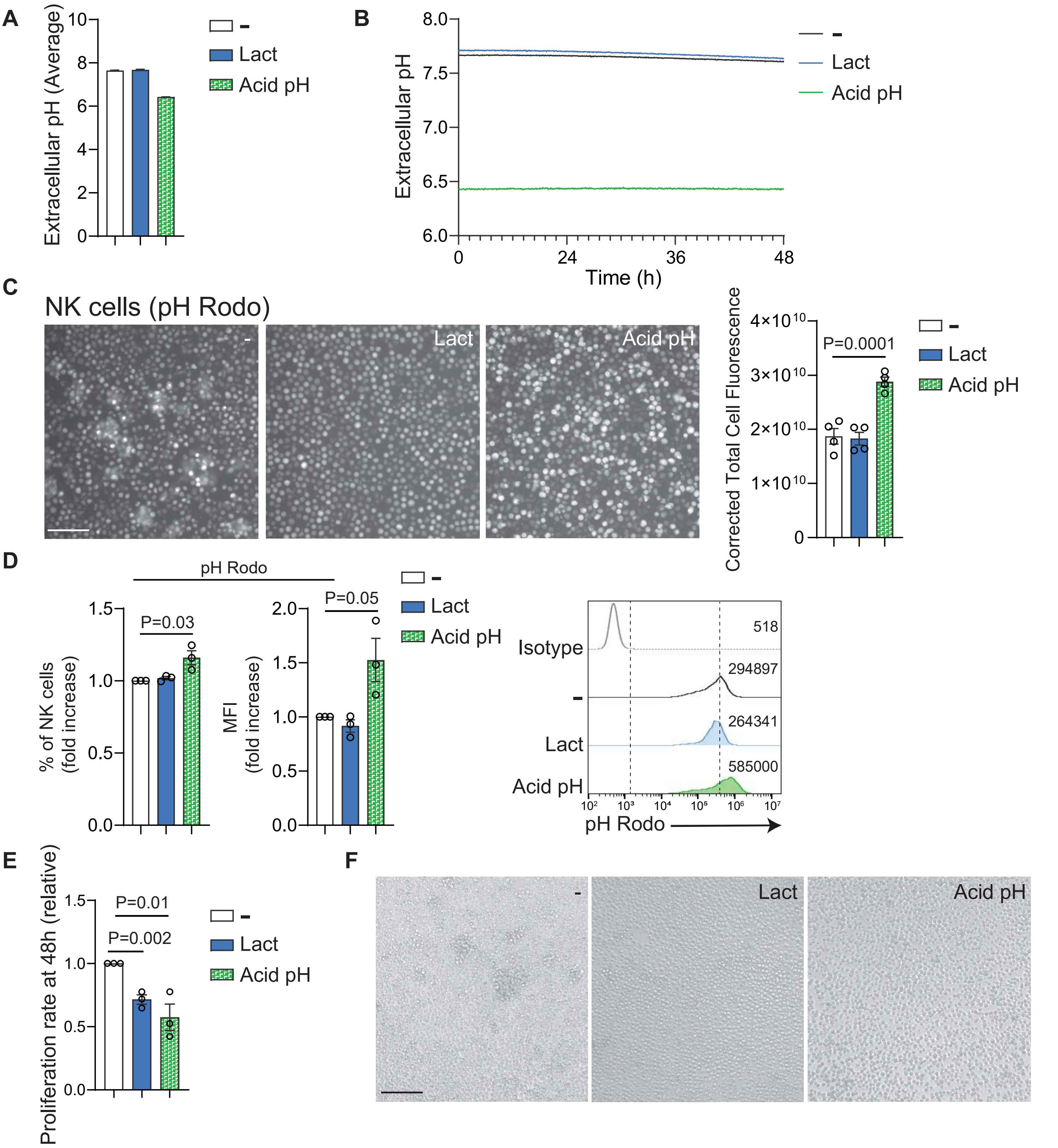

Supplementary figure S3. Lactate modulates NK cell function independently of extracellular or intracellular acidification. A. Average extracellular pH of NK cells cultured under the indicated conditions (control medium (-), lactate (Lact), or HCl-adjusted medium (Acid pH)), measured using PyroScience pH-sensitive sensors. Bars represent mean ± SEM from three independent experiments (n=3). **B**. Representative extracellular pH traces over 48 hours for NK cells cultured under the indicated conditions (n=1), monitored using PyroScience pH-sensitive sensors. **C**. Representative images of NK cells showing intracellular pH, visualized with pHrodo staining, following the indicated treatments. Bars on the right represent mean ± SEM from four independent NK cell donors (n=4). **D**. FACS analysis of intracellular pH (pHrodo) in NK cells cultured under the indicated conditions, shown as percentage of positive cells (left) and MFI fold change (right). Representative flow cytometry histograms of MFI are also shown. Data represent mean ± SEM from three independent NK cell donors (n=3). **E**. NK cell proliferation rate after 48 hours of incubation under the indicated conditions. Data represent mean ± SEM from three independent NK cell donors (n=3). In C-E, each dot represents NK cells from a different healthy donor. **F**. Representative bright-field images of NK cells cultured under the indicated conditions for 48 hours. Statistical analysis was performed using one-way ANOVA followed by Dunn's multiple-comparison test. Exact p-values are reported for statistically significant comparisons; comparisons not shown were not significant.

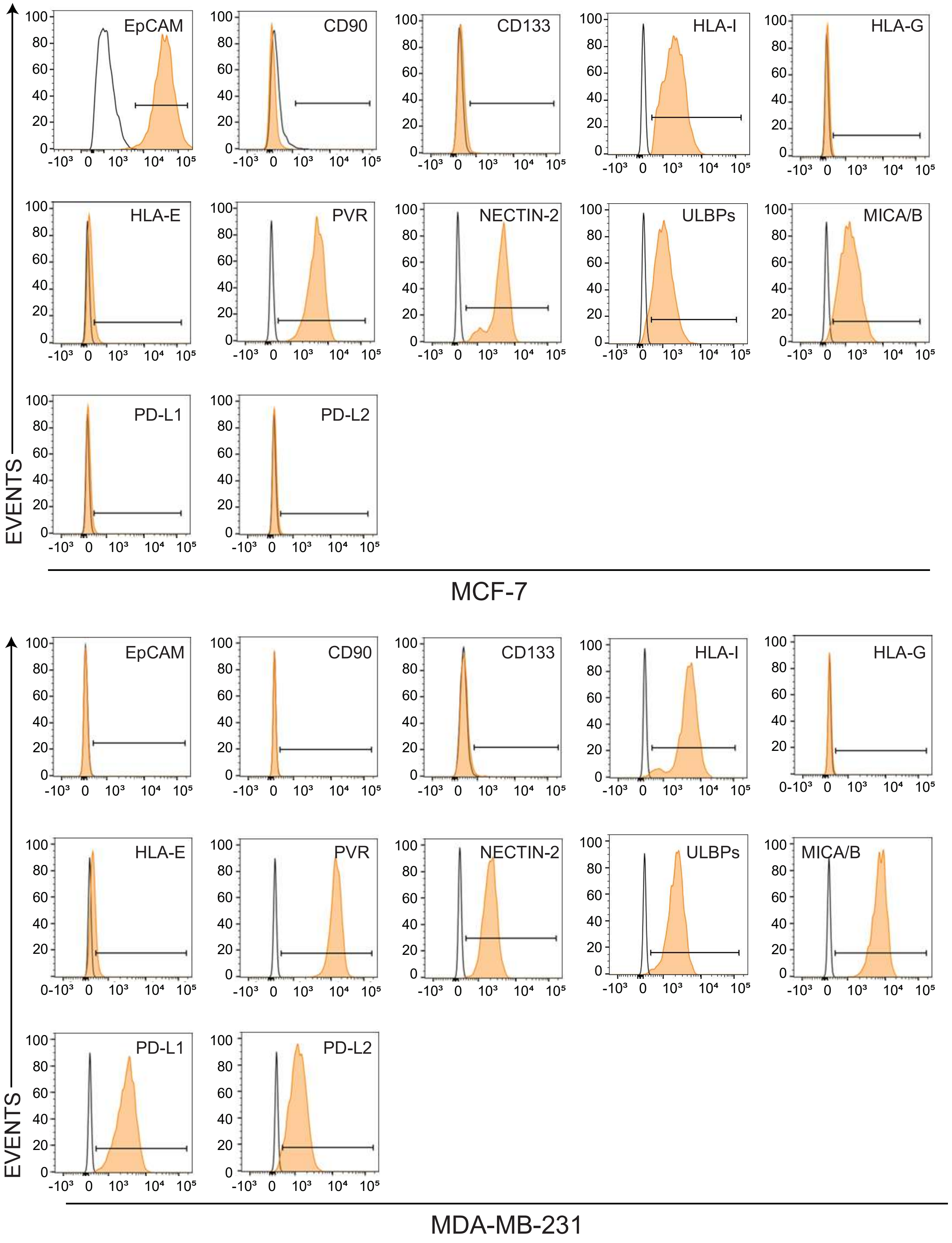

Supplementary figure S4 related to Figure 4. Flow cytometric analysis of surface marker expression in MCF-7 and MDA-MB-231 cells. Representative flow-cytometry histograms of the indicated markers in the MCF-7 (top) and MDA-MB-231 (bottom) breast cancer cell line. For each marker, the specific antibody staining (solid line, filled area) is overlaid with the corresponding isotype control (solid line, empty area).

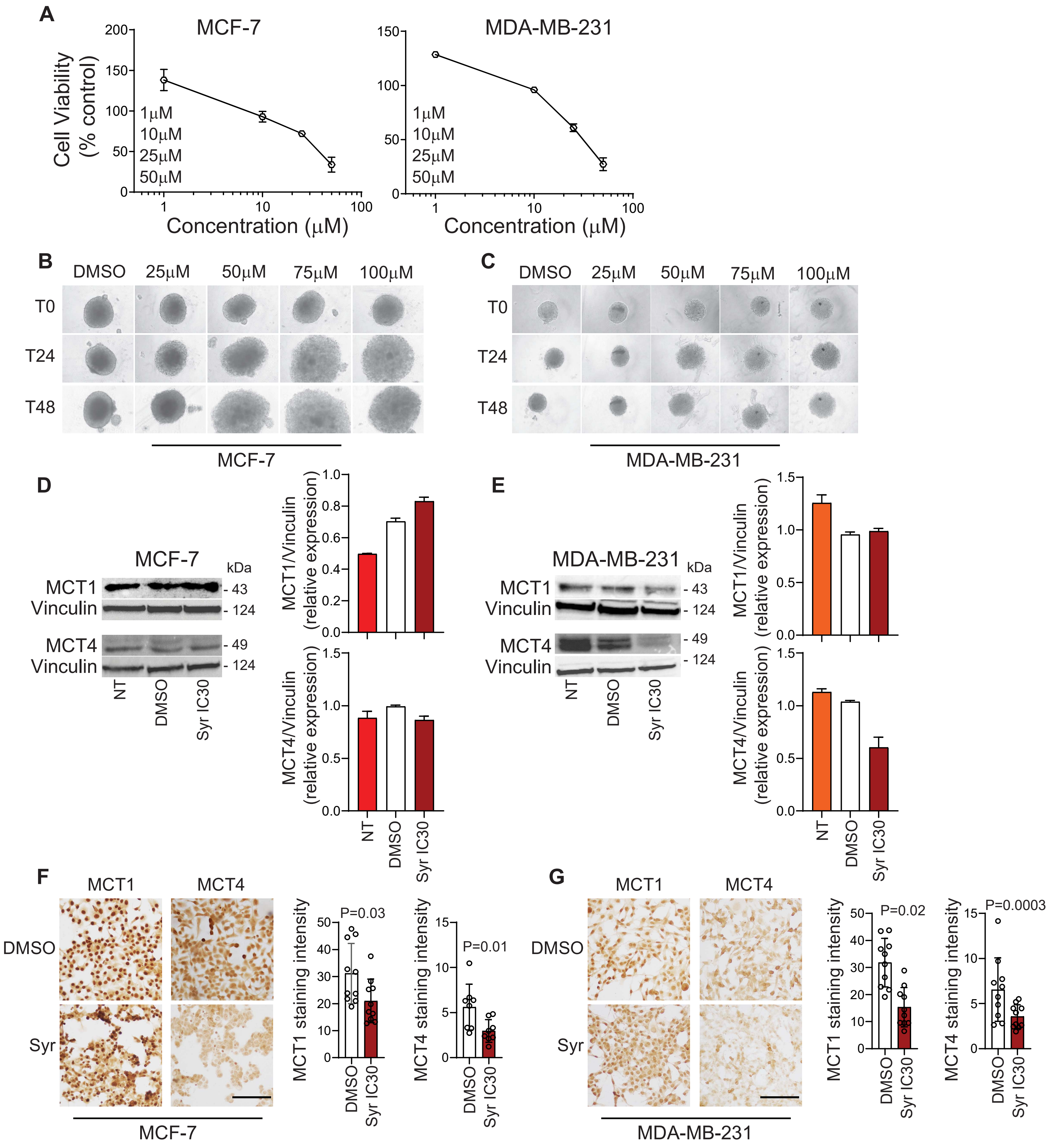

Supplementary figure S5 related to Figure 5. Effect of syrosingopine on cell viability and MCT1 and MCT4 expression in MCF-7 and MDA-MB-231 Cells. A, Dose–response curve for syrosingopine-treated and untreated MCF-7 and MDA-MB-231 cells cultured in 2D, assessed by SRB assay. IC_{30} values were determined by nonlinear regression analysis from three independent experiments. **B**, **C**, Representative images of MCF-7 (B) and MDA-MB-231 (C) cells cultured in 3D tumor spheroids and treated with increasing concentrations of syrosingopine (25, 50, 75, and 100 μM) or DMSO. **D**, **E**, Representative immunoblot analysis of MCT1 and MCT4 expression in (D) MCF-7 and (E) MDA-MB-231 cell lines following IC_{30} syrosingopine treatment. Endogenous expression and DMSO-treated controls are also shown. Densitometric quantification normalized to vinculin from two independent experiments (n=2) is displayed on the right. Data are presented as mean ± SEM. Statistical analysis was not performed due to the low sample size. **F**, **G**, Representative immunocytochemistry images of MCT1 and MCT4 in (F) MCF-7 and (G) MDA-MB-231 cell lines following DMSO or IC_{30} syrosingopine treatment. Positive cells are stained in brown. Original magnification ×20; scale bar, 30 μm. Quantification is shown on the right. Data represent mean ± SEM; each dot represents a different area of the slide (n=10 for all except MCT4 in MCF7 (n = 8)). Statistical significance was assessed using a two-tailed Student's t-test, and exact *p*-values are reported in the individual plots.

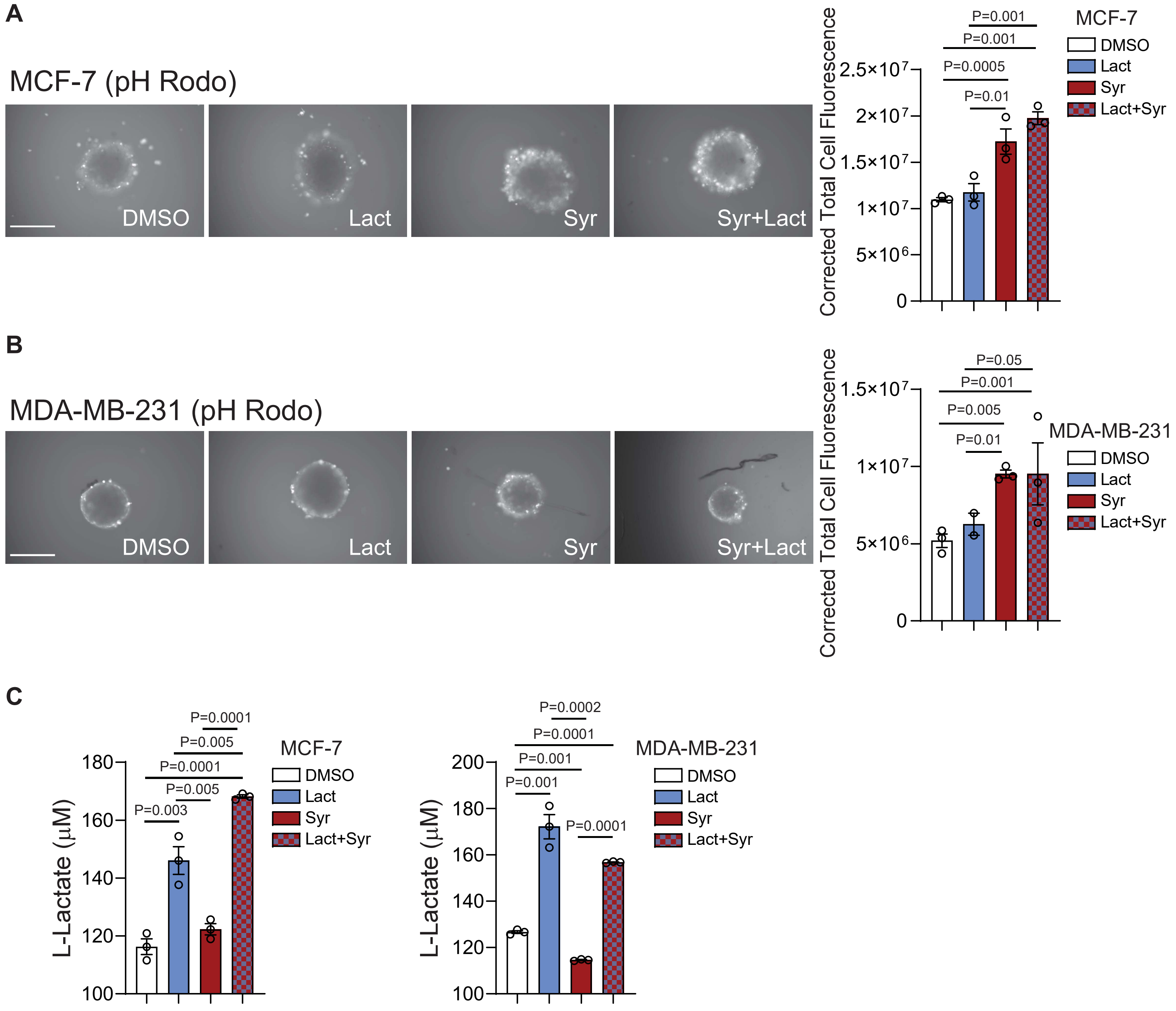

Supplementary figure S6 related to Figure 5. Effects of syrosingopine on intracellular pH and L-Lactate production in MCF-7 and MDA-MB-231 tumor spheroids. A, **B**, Representative images of MCF-7 (A) and MDA-MB-231 (B) tumor spheroids showing intracellular pH, visualized with pHrodo staining, following treatment with DMSO, lactate, IC_{30} syrosingopine, or a combination of lactate and IC_{30} syrosingopine. On the right, bars represent mean ± SEM from three independent experiments performed (n=3). Statistical analysis was performed using one-way ANOVA followed by Dunn's multiple-comparison test. Exact *p*-values are reported where statistically significant; comparisons not indicated did not reach statistical significance. **C**, Measurement of L-lactate production in supernatants from MCF-7 (left) and MDA-MB-231 (right) cell lines following treatment with DMSO, lactate, IC_{30} syrosingopine, or the combination of lactate and IC_{30} syrosingopine. Data represent mean ± SEM from three independent experiments (n=3). Statistical analysis was performed using one-way ANOVA followed by Dunn's multiple-comparison test. Exact *p*-values are reported where statistically significant; comparisons not indicated did not reach statistical significance.

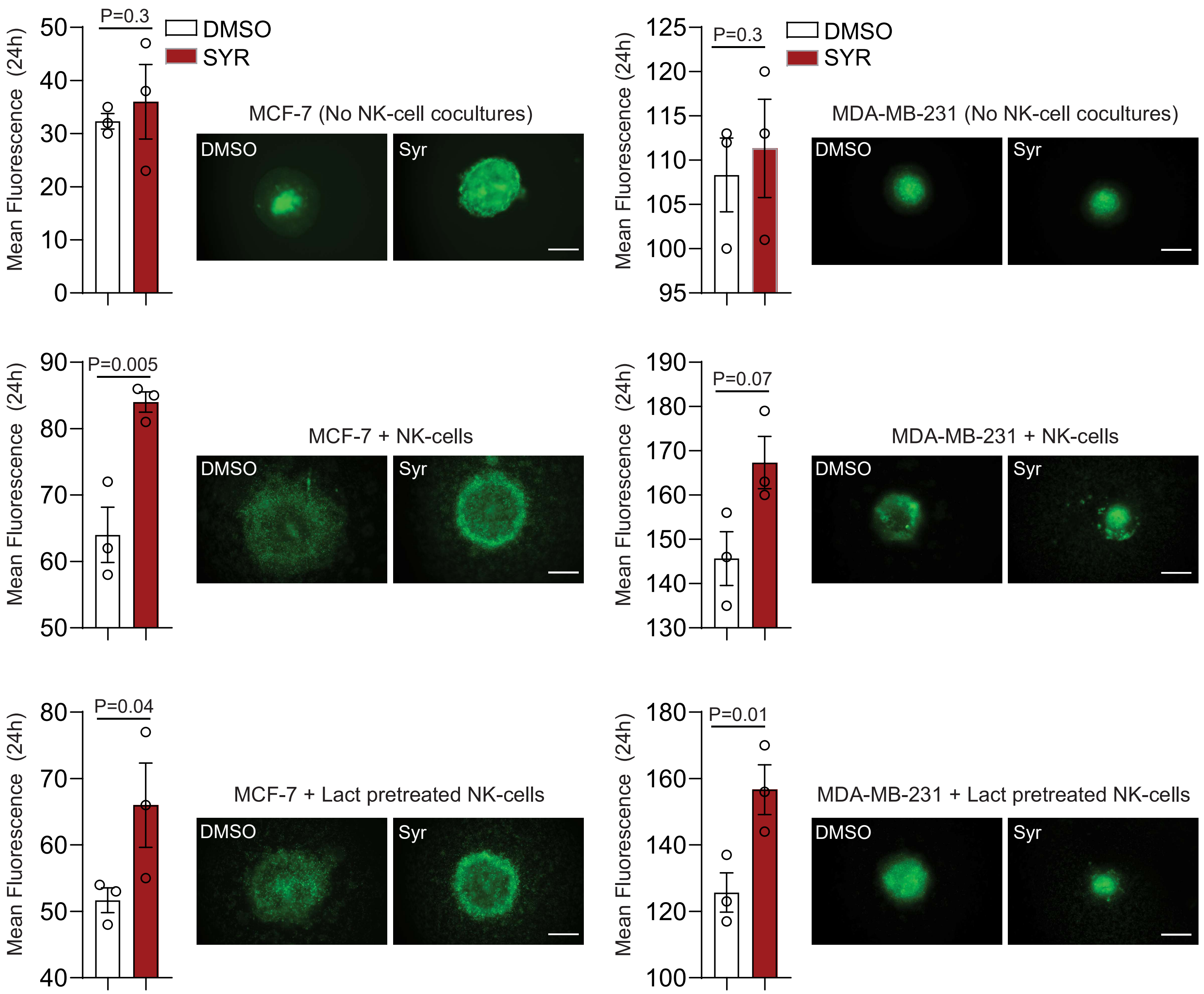

Supplementary figure S7 related to Figure 5. Syrosingopine enhances NK cell–mediated caspase-3/7 activation in Breast Cancer tumor spheroids. Bar charts (left) and representative images (right) of caspase-3/7 activation (green fluorescence) in MCF-7 (left) or MDA-MB-231 (right) tumor spheroids cultured for 24 hours alone (top), with NK cells (middle), or with lactate-exposed NK cells in the presence of DMSO or syrosingopine. Bars represent mean ± SEM from three independent experiments (n=3). Statistical analysis was performed using a two-tailed Student's t-test. Exact *p*-values are reported in the corresponding plots.

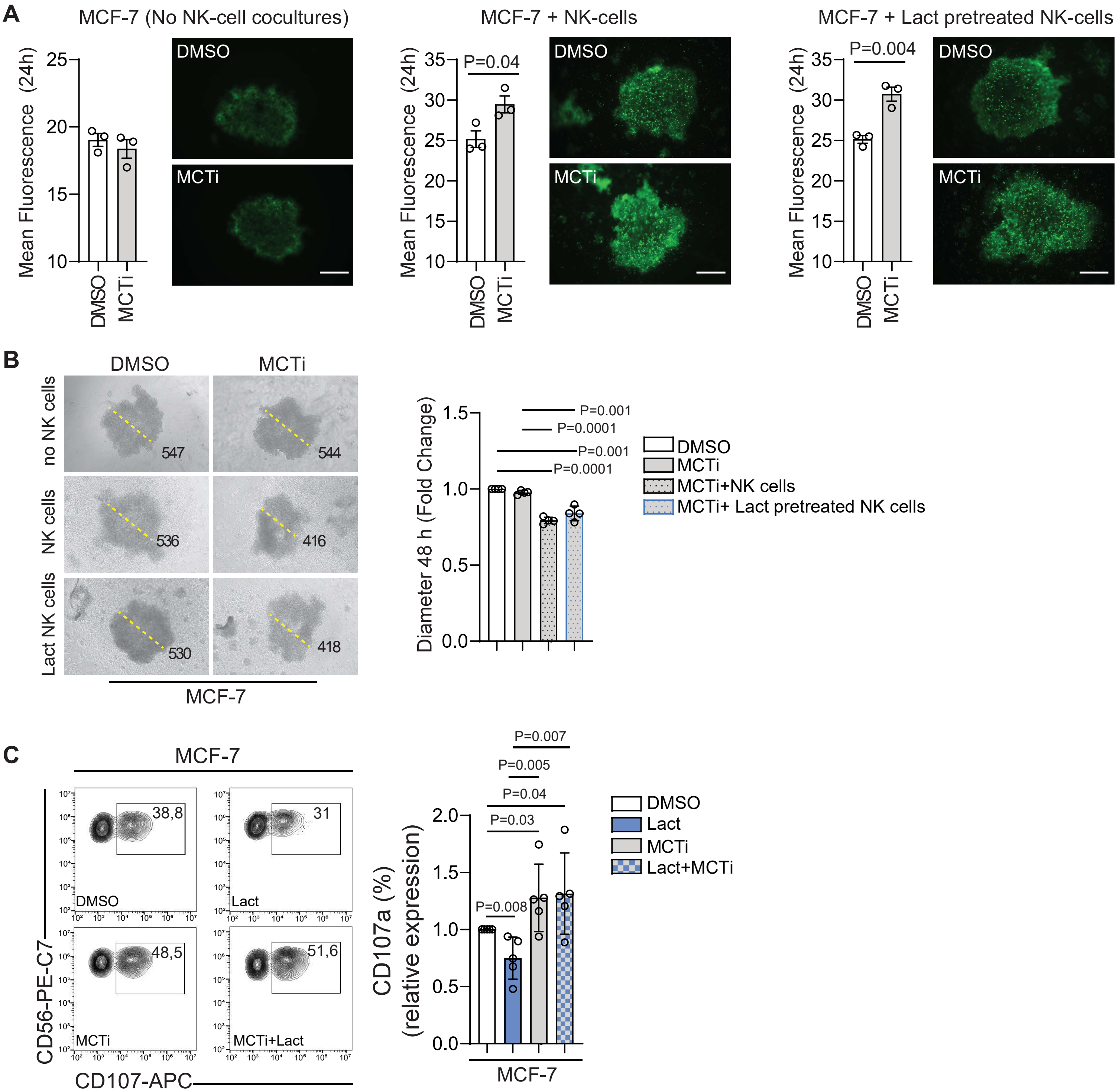

Supplementary figure S8 related to Figure 5. Combined MCT1/4 inhibition recapitulated syrosingopine's effect in MCF-7 cells. A, Bar charts (left) and representative images (right) of caspase-3/7 activation (green fluorescence) in MCF-7 tumor spheroids cultured for 24 hours alone (left), with NK cells (middle), or with lactate-exposed NK cells (right) in the presence of DMSO or AZD3965 (MCT1 inhibitor) and MSC-4381 (MCT4 inhibitor) combination (MCTi). Data are expressed as mean ± SEM and are representative of three independent biological experiments performed with NK cells from three different healthy donors (n=3). Statistical analysis was performed using a two-tailed Student's t-test. **B**, Representative images of diameter measurements in MCF-7 tumor spheroids cultured for 48 hours alone (top), with NK cells (middle), or with lactate-exposed NK cells in the presence of DMSO or MCTi treatment. On the right, bar graphs represent mean ± SEM from independent biological experiments performed with NK cells from four different healthy donors (n=4). Statistical analysis was performed using one-way ANOVA followed by Dunn's multiple-comparison test. **C,** Representative example of degranulation by human $CD45^+CD56^+CD16^+CD3^-$ NK cells from a healthy donor, measured as CD107a cell-surface expression following stimulation with MCF-7 target cells in the presence of DMSO, lactate (Lact), MCTi (0.1 µM), or the combination of lactate with MCTi (Lact + 0.1 µM MCTi). The percentage of $CD107a^+$ NK cells is indicated. On the right, summary of NK cell degranulation using NK cells from five healthy donors under the same conditions. Statistical analysis was performed using one-way ANOVA followed by Dunn's multiple comparison test. In **A-C**, each dot in bar chart represents NK cells from a different healthy donor and exact *p*-values are reported in the corresponding plots where statistically significant.

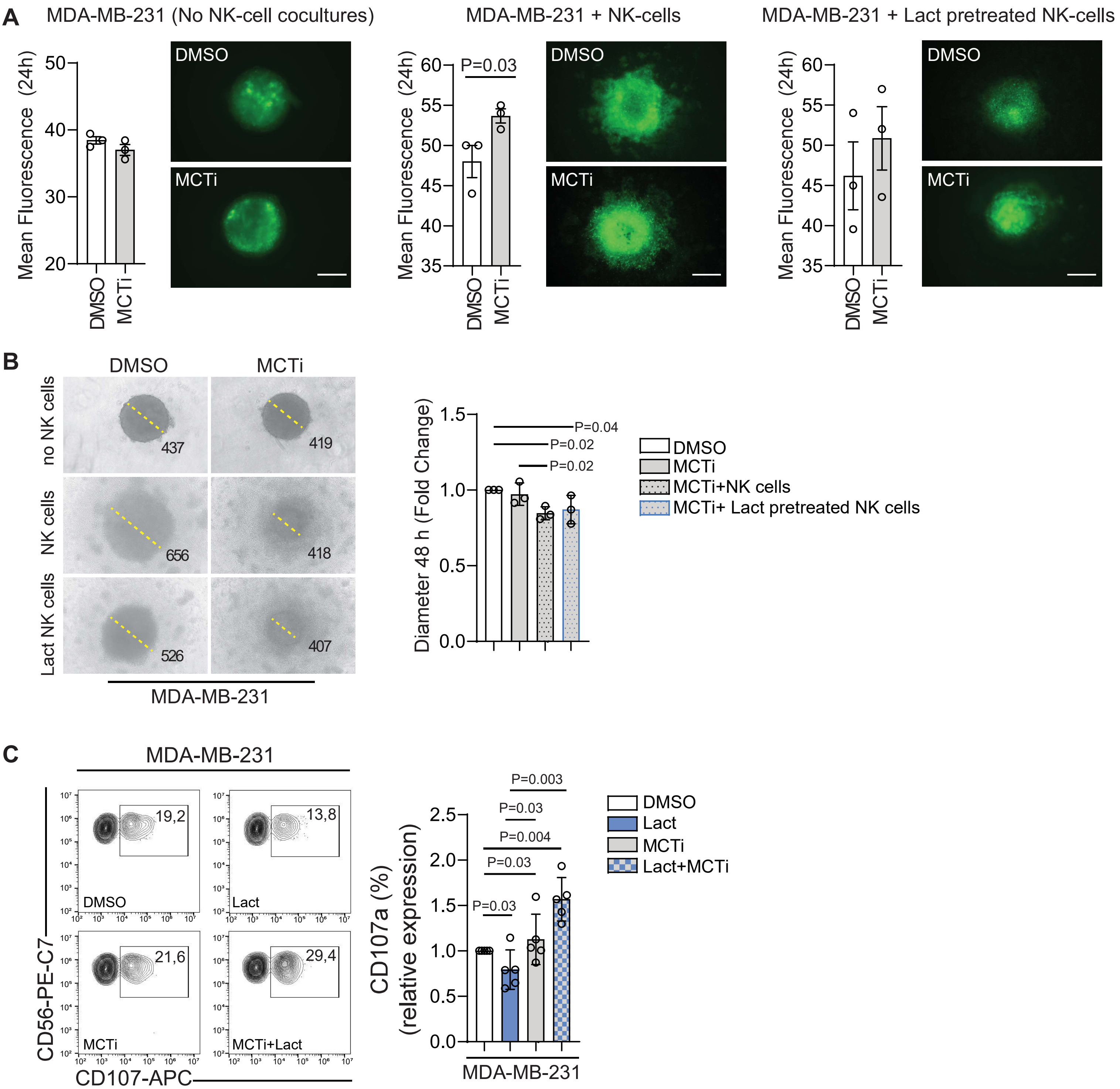

Supplementary figure S9 related to Figure 5. Combined MCT1/4 inhibition recapitulated syrosingopine's effect in MDA-MB-231 cells. A, Bar charts (left) and representative images (right) of caspase-3/7 activation (green fluorescence) in MDA-MB-231 tumor spheroids cultured for 24 hours alone (left), with NK cells (middle), or with lactate-exposed NK cells (right) in the presence of DMSO or AZD3965 (MCT1 inhibitor) and MSC-4381 (MCT4 inhibitor) combination (MCTi). Data are expressed as mean ± SEM and are representative of three independent biological experiments performed with NK cells from three different healthy donors (n=3). Statistical analysis was performed using a two-tailed Student's t-test. **B**, Representative images of diameter measurements in MDA-MB-231 tumor spheroids cultured for 48 hours alone (top), with NK cells (middle), or with lactate-exposed NK cells in the presence of DMSO or MCTi treatment. On the right, bar graphs represent mean ± SEM from three independent biological experiments performed with NK cells from three different healthy donors (n=3). Statistical analysis was performed using one-way ANOVA followed by Dunn's multiple-comparison test. **C,** Representative example of degranulation by human $CD45^{+}CD56^{+}CD16^{+}CD3^{-}$ NK cells from a healthy donor, measured as CD107a cell-surface expression following stimulation with MDA-MB-231 target cells in the presence of DMSO, lactate (Lact), MCTi (0.1 μM), or the combination of lactate with MCTi (Lact + 0.1 μM MCTi). The percentage of $CD107a^{+}$ NK cells is indicated. On the right, summary of NK cell degranulation using NK cells from five healthy donors under the same conditions. Statistical analysis was performed using one-way ANOVA followed by Dunn's multiple comparison test. In **A-C**, each dot in bar chart represents NK cells from a different healthy donor and exact *p*-values are reported in the corresponding plots where statistically significant.

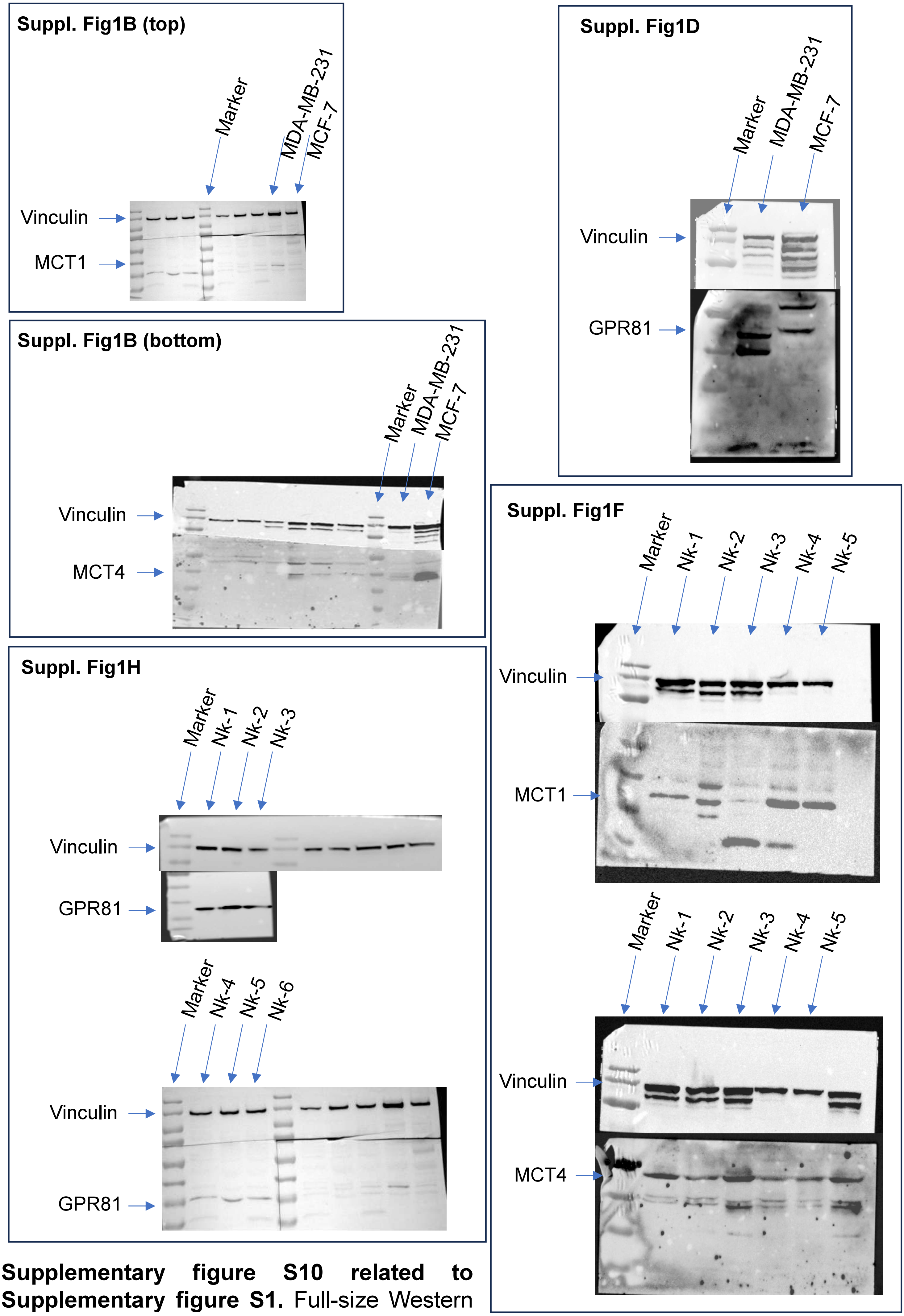

Supplementary figure S10 related to Supplementary figure S1. Full-size Western blots and high-resolution imaging data.

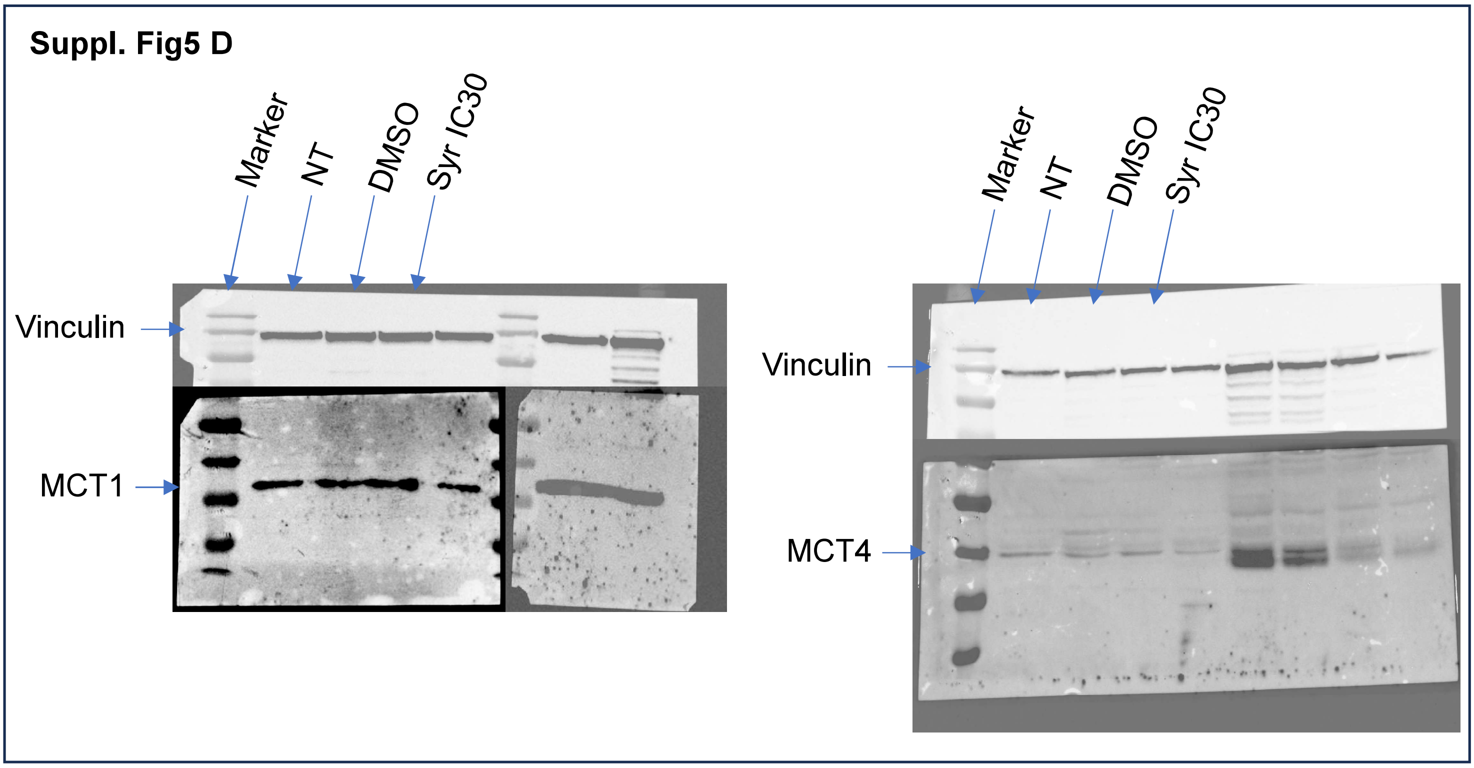

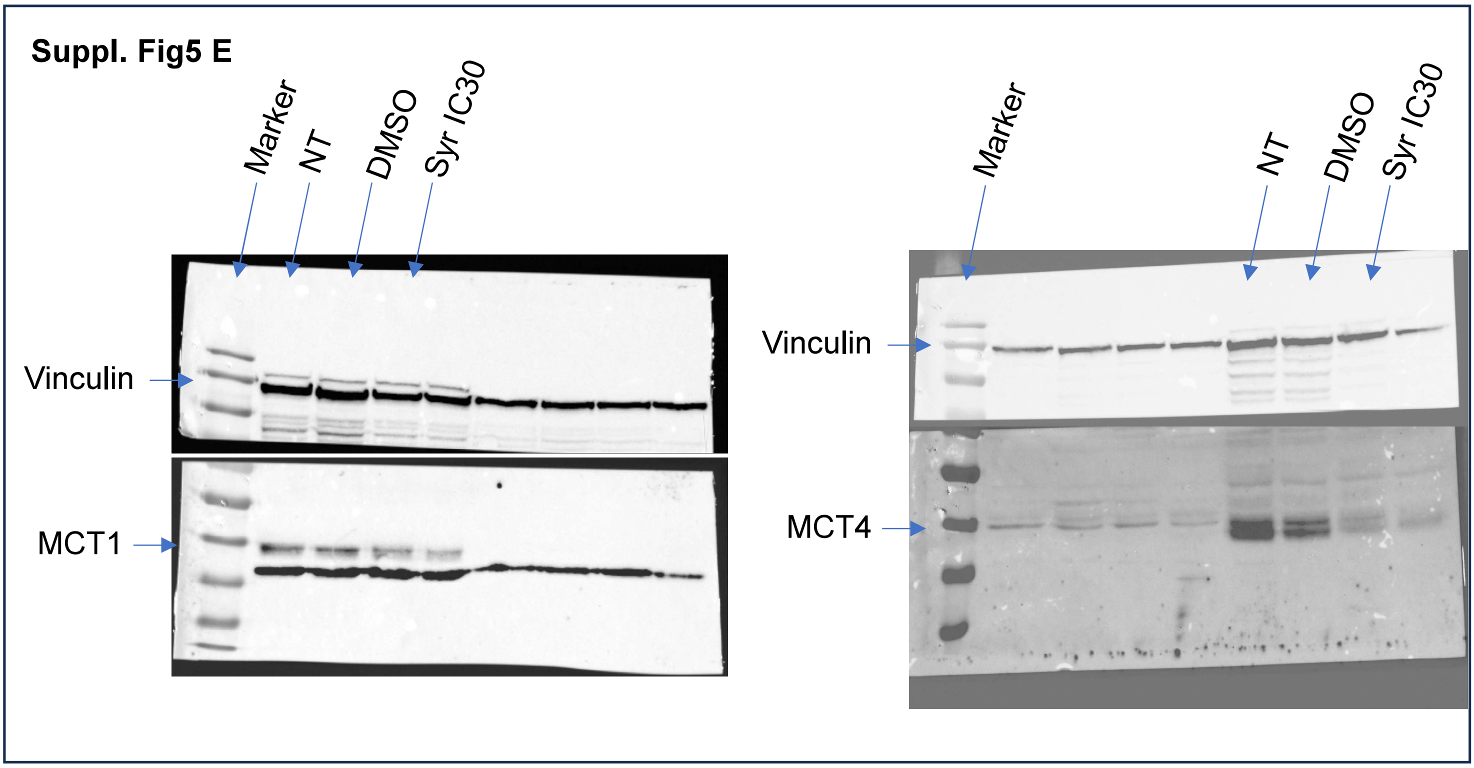

Supplementary figure S11 related to Supplementary figure S5. Full-size Western blots and high-resolution imaging data.
