# Supplementary material for: Lactate-mediated NK cell dysfunction as a prognostic marker and therapeutic target in breast cancer
Source: Cell Death Discov. 2026 Mar 27;12:200. doi: 10.1038/s41420-026-03063-5 (PMC13149829; doi:10.1038/s41420-026-03063-5)
Supplement: Supplementary file 2 — Supplementary Tables [file 41420_2026_3063_MOESM2_ESM.pdf]

Gene(s)	Model	Comparison	Effect estimate	95% CI	P value
HCAR1	Cox PH	HCAR1^{Lo} vs HCAR1^{Hi}	HR = **0.68**	[0.59, 0.79]	$3.8x10^{-7}$
LDHA	RMST (τ =31.9)	LDHA ^{Hi} vs LDHA ^{Lo}	ΔRMST=-3.00 yrs	[-3.7, -2.29]	$1.11x10^{-16}$
NCR1	RMST (τ =39.7)	NCR1^{Hi} vs NCR1^{Lo}	ΔRMST=1.61 yrs	[0.89, 2.32]	$1.06x10^{-5}$
NCR2	RMST (τ =31.9)	NCR2^{Hi} vs NCR2^{Lo}	ΔRMST=2.02 yrs	[1.29, 2.76]	$5.55x10^{-8}$
NCR3	RMST (τ =31.9)	NCR3^{Hi} vs NCR3^{Lo}	ΔRMST=2.41 yrs	[1.56, 3.27]	$3.53x10^{-8}$
HCAR1-NCR1	Cox PH	HCAR1^{Hi}-NCR1^{Lo} vs HCAR1^{Lo} -NCR1^{Hi}	HR=1.81	[1.51, 2.18]	$2.6x10^{-10}$
HCAR1-NCR2	Cox PH	HCAR1^{Hi} -NCR2^{Lo} vs HCAR1^{Lo} -NCR2^{Hi}	HR=1.88	[1.56, 2.27]	$5.3x10^{-11}$
		HCAR1^{Hi} -NCR2^{Hi} vs HCAR1^{Lo} -NCR2^{Hi}	HR=1.41	[1.03, 1.92]	$3.3 x10^{-2}$
		HCAR1^{Lo} -NCR2^{Lo} vs HCAR1^{Lo} -NCR2^{Hi}	HR=1.38	[1.17, 1.63]	$1.1 x10^{-4}$
HCAR1-NCR3	RMST (τ =24.4)	HCAR1^{Lo} -NCR2^{Hi} vs HCAR1^{Hi} -NCR3^{Lo}	ΔRMST=3.19 yrs	[2.22, 4.16]	$1.15x10^{-10}$
		HCAR1^{Hi} -NCR2^{Hi} vs HCAR1^{Hi} -NCR3^{Lo}	ΔRMST=1.96 yrs	[-0.12, 4.04]	$6.5 x10^{-2}$
		HCAR1^{Lo} -NCR2^{Lo} vs HCAR1^{Hi} -NCR3^{Lo}	ΔRMST=1.23 yrs	[0.46, 1.97]	$1.2x10^{-3}$
LDHA-NCR1	RMST (τ =24.4)	LDHA^{Hi} -NCR1^{Hi} vs LDHA^{Hi} -NCR1 ^{Lo}	ΔRMST=1.83 yrs	[0.87, 2.79]	$1.8 x10^{-4}$
		LDHA ^{Lo} -NCR1^{Hi} vs LDHA^{Hi} -NCR ^{Lo}	ΔRMST=2.96 yrs	[2.14, 3.77]	$1.5x10^{-12}$
		LDHA ^{Lo} -NCR1 ^{Lo} vs LDHA^{Hi} -NCR1 ^{Lo}	ΔRMST=3.62 yrs	[2.71, 4.54]	$7.8x10^{-15}$
LDHA-NCR2	RMST (τ =24.4)	LDHA^{Hi} -NCR2^{Hi} vs LDHA^{Hi} -NCR2 ^{Lo}	ΔRMST=2.07 yrs	[1.08, 3.06]	$3.8x10^{-5}$
		LDHA ^{Lo} NCR2^{Hi} vs LDHA^{Hi} -NCR2 ^{Lo}	ΔRMST=3.34 yrs	[2.51, 4.18]	$3.9x10^{-15}$
		LDHA ^{Lo} NCR2 ^{Lo} vs LDHA^{Hi} -NCR2 ^{Lo}	ΔRMST=3.17 yrs	[2.29, 4.05]	$1.3x10^{-12}$
LDHA-NCR3	RMST (τ =24.4)	LDHA^{Hi} -NCR3^{Hi} vs LDHA^{Hi} -NCR3 ^{Lo}	ΔRMST=2.11 yrs	[0.78, 3.45]	$1.9 x10^{-3}$
		LDHA ^{Lo} -NCR3^{Hi} vs LDHA^{Hi} -NCR3 ^{Lo}	ΔRMST=3.60 yrs	[2.67, 4.53]	$3.0x10^{-14}$
		LDHA ^{Lo} -NCR3 ^{Lo} vs LDHA^{Hi} -NCR3 ^{Lo}	ΔRMST=2.69 yrs	[1.90, 3.48]	$2.0x10^{-11}$

Supplementary Table 1. Multivariable survival analyses evaluating the association between *HCAR1* or *LDHA* expression and *NCR1, NCR2,* and *NCR3* with recurrence-free survival. All models were adjusted for age at diagnosis, tumor grade, HER2 status, estrogen receptor (ER) status, and tumor size. Cox proportional hazards (Cox PH) models were applied when the proportional hazards assumption was satisfied, while restricted mean survival time (RMST) analyses were used otherwise. Results are reported as hazard ratios (HRs) or differences in RMST, as appropriate. τ indicates the selected truncation time for RMST estimation.

Antigen	Isotype control	Fluorochrome	Clone	Company	Dilution	Cat.no.	Use
Anti-human CD107a	Mouse IgG1	FITC	H4A3	BD Biosciences	1:12.5	560949	FACS
Anti-human CD3	Mouse IgG1	AlexaFluor-700	UCHT1	BD Biosciences	1:50	557943	FACS
Anti-human CD56	Mouse IgG1	PE-Cy7	B159	BD Biosciences	1:40	557747	FACS
Anti-human CD16	CDF1 IgG1	PE	3G8	BD Biosciences	1:50	555407	FACS
Annexin-V	-	PE	-	Biolegend	1:20	640908	FACS
Anti-human GZBM	Mouse IgG1	BV510	GB11	BD Biosciences	1:50	563388	FACS
Anti-human IFNg	Rat IgG2a	PE	4S. B3	Sony	1:50	287849	FACS
Anti-human CD16	CDF1 IgG1	PerCP-Cy5.5	3G8	BD Biosciences	1:200	560717	FACS
Anti-human NKG2A	Mouse IgG1	PE-Cy7	Z199	Beckman Coulter	1:40	B10246	FACS
Anti-human NKp30	Mouse IgG1	BV605	p30-15	BD Biosciences	1:10	563384	FACS
Anti-human NKp44	Mouse IgG1	BV650	p44-8	BD Biosciences	1:25	744302	FACS
Anti-human NKp46	Mouse IgG1	PE	REA808	Miltenyi	1:25	130-112-121	FACS
Anti-human CD69	Mouse IgG1	BUV737	FN50	BD Biosciences	1:40	612817	FACS
Anti-human CD25	Mouse IgG2a	FITC	B1.49.9	Beckman Coulter	1:10	IM0478U	FACS
Anti-human CD57	Mouse IgM	BUV395	NK-1	BD Biosciences	1:100	567621	FACS
Anti-human KIR3DL1	Mouse IgG1	PE	DX9	Miltenyi	1:20	130-092-473	FACS
Anti-human KIR2DL2	Mouse IgG2b	FITC	CH.-L	BD Biosciences	1:12,5	559784	FACS
Anti-human EPCAM	Mouse IgG1	APC-Vio770	REA764	Miltenyi	1:40	130-111-002	FACS
Anti-human CD90	Mouse IgG1	PE-Vio770	REA897	Miltenyi	1:25	130-114-862	FACS
Anti-human CD133	Mouse IgG1	APC	REA753	Miltenyi	1:50	130-110-963	FACS
Anti-human HLA-I	Mouse IgM	Not conjugated	A6136	#	1:50	#	FACS
Anti-human HLA-G	Mouse IgG1	Not conjugated	MEM-G/9	Abnova	1:50	MAB3855	FACS
Anti-human HLA-E	Mouse IgG1	Not conjugated	3D12	Biolegend	1:50	342602	FACS
Anti-human PVR	Mouse IgG1	Not conjugated	5A10	#	1:50	#	FACS
Anti-human NECTIN	Mouse IgG2a	Not conjugated	L14	#	1:50	#	FACS
Anti-human ULBP1	Mouse IgG2a	Not conjugated	170818	R&D System	1:50	MAB1380	FACS
Anti-human ULBP2	Mouse IgG2a	Not conjugated	165903	R&D System	1:50	MAB1298	FACS
Anti-human ULBP3	Mouse IgG2a	Not conjugated	166510	R&D System	1:40	MAB1517	FACS

Anti-human ULBP4	Mouse IgG2b	Not conjugated	6E6	Santa Cruz Biotechnologies	1:40	sc-53133	FACS
Anti-human MIC-A/B	Mouse IgG1	Not conjugated	BAM195	#	1:50	#	FACS
Anti-human PD-L1	Mouse IgG1	APC-R700	MIH1	BD	1:40	565188	FACS
Anti-human PD-L2	Mouse IgG1	APC-R700	MIH18	BD	1:30	565189	FACS
Isotype-specific goat anti-mouse IgM	-	PE	-	Southern Biotechnology	1:200	1020-09	FACS
Isotype-specific goat anti-mouse IgG1	-	PE	-	Southern Biotechnology	1:200	1070-09	FACS
Isotype-specific goat anti-mouse IgG2a	-	PE	-	Southern Biotechnology	1:200	1080-09	FACS
APC-R700 Mouse IgG1, κ Isotype Control	-	APC-R700	-	BD Biosciences	1:200	564974	FACS
Anti-human MCT-1	Mouse IgG1	Not conjugated	H-1	Santa Cruz Biothecnologies	1:100	Sc-365501	IHC, WB
Anti-human MCT-4	Mouse IgG2a	Not conjugated	D-1	Santa Cruz Biothecnologies	1:100	Sc-376140	IHC
Anti MCT-4/SLC16A3	Rabbit IgG	Not conjugated	polyclonal	ABclonal	1:500	A10548	WB
Anti-human GPR81	Mouse IgG	Not conjugated	1048103	R&D System	1:100	MAB1113 4	WB
Anti-human Vinculin	Mouse IgG	Not conjugated	hVIN-1	Sigma-Aldrich	1:50000	V9131	WB
Goat Anti-Mouse IgG	-	(H+L)-HRP	-	Bio-Rad	1:5000	1706516	WB
Goat Anti-Rabbit IgG	-	(H+L)-HRP	-	Bio-Rad	1:10000	1706515	WB

Supplementary Table 2: List of primary and secondary antibodies

Isolated from Molecular Immunology Laboratory, DIMES, Genoa (Italy);
FACS: Flow Cytometry, IHC: immunohistochemistry; WB: Western Blotting.
During the experiments, batches were changed and compared with the previous batch to confirm dilution.
